# Generation and Characterization of Novel Contilisant+Tubastatin a Multitarget Small Molecules Against Glioblastoma

**DOI:** 10.3390/pharmaceutics17121594

**Published:** 2025-12-10

**Authors:** Irati de Goñi, Aizpea Artetxe-Zurutuza, Joseba Elizazu, Raul Garcia-Garcia de Garayo, Jhonatan Vergara-Arce, Mikel Azkargorta, Mireia Toledano-Pinedo, Alicia Porro-Pérez, Felix Elortza, Jose Luis Marco-Contelles, Nicolas Sampron, Nerea Iturrioz-Rodriguez, Ander Matheu

**Affiliations:** 1 Cellular Oncology Group, Biogipuzkoa (Biodonostia) Health Research Institute, 20014 San Sebastian, Spain; 2 Neurosurgery Service, Donostia University Hospital, 20014 San Sebastian, Spain; 3 Proteomics Platform, CIC bioGUNE, Basque Research and Technology Alliance (BRTA), CIBERehd, ProteoRed-ISCIII, 48160 Derio, Spain; 4 Laboratory of Medicinal Chemistry, Institute of General Organic Chemistry (CSIC), 28006 Madrid, Spain; 5 Centre for Biomedical Network Research on Rare Diseases (CIBERER), Instituto de Salud Carlos III (ISCIII), 28029 Madrid, Spain; 6 Centre for Biomedical Network Research on Frailty and Healthy Aging (CIBERFES), Instituto de Salud Carlos III (ISCIII), 28029 Madrid, Spain; 7 IKERBASQUE, Basque Foundation for Science, 48009 Bilbao, Spain

**Keywords:** glioblastoma, MSM, HDAC inhibition, glioma stem cells, proliferation

## Abstract

**Background/Objectives**: Glioblastoma is the most common and aggressive primary brain tumor in adults, with patient prognosis remaining poor. Treatment resistance and tumor recurrence are frequent, primarily due to the high intra- and inter-tumoral heterogeneity and the existence of glioma stem cells. Thus, there is an urgent need for novel and more effective therapeutic strategies. Multitarget small molecules (MSMs) are emerging as a novel therapeutic strategy for the treatment of complex diseases such as cancer. **Methods**: In the present work, we have generated a novel family of indole-based MSMs with pharmacophoric moieties combining the parent compounds Contilisant and the HDAC inhibitor Tubastatin A. Thus, the MSMs were designed to inhibit monoamine oxidases (MAOs), cholinesterases (ChEs) and histone deacetylases (HDACs), while acting as histamine H3 receptor (H3R) antagonists and sigma 1 receptor (S1R) agonists. We generated four different molecules and evaluated in detail the activity of the two most efficient MSM compounds in vitro and in vivo. **Results**: These molecules induced potent cytotoxic effects in vitro in patient-derived glioma stem cells and glioblastoma cell lines and significantly impaired tumor growth in vivo. OMIC analyses further revealed that the compounds induce dysregulation of the cell cycle in glioma stem cells. Moreover, in silico analyses indicated that these compounds are theoretically capable of crossing the blood–brain barrier, while exhibiting low toxicity in healthy cells. **Conclusions**: In conclusion, our findings demonstrate the potential antitumor activity of a novel family of MSMs in preclinical models of glioblastoma.

## 1. Introduction

Gliomas are the most common and malignant primary brain tumors in adults, representing almost 30% of all primary brain tumors and accounting for 75–80% of malignancies. In terms of morbidity, the majority of deaths from primary brain tumors are caused by gliomas [1,2]. Glioblastoma is a grade IV glioma, being the most prevalent and aggressive primary brain tumor, with patient prognosis remaining exceedingly poor [3]. GBM presents an overall survival of around 15 months and a 5-year survival rate of less than 5% [3]. The current standard of care involves surgical resection followed by radiotherapy and chemotherapy with temozolomide (TMZ) [4]. Despite this multimodal approach, treatment resistance and tumor recurrence are frequent, primarily due to the high intra- and inter-tumoral heterogeneity, the presence of glioma stem cells (GSCs) and the complexity of the tumor microenvironment [5,6]. Consequently, there remains an urgent need for novel and more effective therapeutic strategies.

Over the past few decades, drug development has largely focused on designing highly selective single-target compounds, aiming to minimize off-target effects and reduce adverse outcomes [7]. However, growing insights into the molecular underpinnings of complex diseases including neurodegenerative diseases and cancer have revealed that these pathologies often involve the dysregulation of multiple signaling pathways. As a result, single-target therapies frequently fall short in effectively addressing such multifactorial diseases [8]. In this context, the multitarget approach has gained significant traction in recent years as a promising strategy in drug discovery. Several compounds and therapeutic modalities have been developed since then, demonstrating enhanced therapeutic efficacy with a more favorable side-effect profile, particularly in the context of cancer treatment [9,10]. One example of a successful multitarget drug is entrectinib, a small molecule that inhibits TRK, ROS1 and ALK. In fact, this orally administered compound has been approved for the treatment of locally advanced or metastatic solid tumors with NTRK1/2/3, ROS1 and ALK gene fusion mutations [8]. Multitarget directed compounds display additional advantages [11]. The pharmacokinetic and pharmacodynamic relationships are more predictable [12]. Further, even if the activity exerted by multitarget drugs on each of their single targets may be lower than that of single-target compounds’, multitarget regulation can present synergistic effects that ultimately lead to better therapeutic effects and smaller adverse reactions [8]. Additionally, and from a practical point of view, the use of a unique multitarget compound facilitates proper compliance to the treatment schedule by patients [11].

Indole is a naturally occurring heterocyclic compound that exhibits good bioavailability and a wide range of pharmacological activities, making it a promising scaffold for the design and development of new drugs targeting various diseases. Indeed, indole derivatives have been shown to participate in the regulation of biological pathways involved in cancer progression, including cell cycle, tumor vascularization, DNA repair and cell death [13]. Indeed, several indole-based agents have already been approved for clinical use for cancer treatment by the FDA (e.g., osimertinib, alectinib and sunitinib), including for glioblastoma [14].

Contilisant is an indole-based compound that has been synthesized as a multitarget small molecule (MSM). Indeed, it has been described to inhibit monoamine oxidases (MAOs) and cholinesterases (ChEs), as well as modulate histamine 3 receptor (H3R) and sigma 1 receptor (S1R). Contilisant was originally generated for the treatment of neurodegenerative diseases, in which it was demonstrated to be neuroprotective, nontoxic, antioxidant and permeable, and able to restore the cognitive deficits induced by Aβ1-42 in Alzheimer’s disease in vivo [15]. In this context, it is well known that neurodegenerative diseases share pathological mechanisms with cancer. In both cases, pathways related to cell proliferation, apoptosis and DNA repair proteins are known to be altered. Further, they also share common risk factors, including chronic inflammation [16]. Thus, given the complexity of neurodegenerative diseases and cancer and the similarities between them, it has been proposed that the same therapeutic compound could be beneficial for both cases [16]. Thus, the expression of MAOs and ChEs as well as the activity of H3R and S1R are increased in cancer, including glioblastoma [9].

Aberrant expression of HDACs is observed in several types of both solid and hematological tumors. In fact, high expression and deregulated activity of HDACs have been associated with advanced stages of the disease and linked to poor outcome for patients in most cases [17], including in glioblastoma [18,19,20,21]. Among these, HDAC6 is particularly relevant. Moreover, knockdown and overexpression experiments have shown that HDAC6 controls hallmark features of glioblastoma, which include proliferation, invasion and migration, as well as angiogenesis and apoptosis, and the maintenance of the glioma stem cell population [18]. In this context, HDAC6 pharmacological inhibition has been shown to decrease cell proliferation and migration in glioma cells, reduce the activity of glioma stem cells and reduce the growth of both TMZ-sensitive and TMZ-resistant cells in vivo [22]. Tubastatin A is the most effective and promising specific HDAC6 inhibitor against cancer and it has shown antitumor activity in glioblastoma [23].

It is possible to make structural modifications and incorporate new pharmacophoric groups with Contilisant to add novel targets. We have generated different compounds combining Contilisant with the Tubastatin A HDAC6 inhibitor. Thus, in the present study, the potential antitumor effect of a novel family of indole-based MSMs generated by the combination of Contilisant with Tubastatin A is reported in different cancers, focusing on glioblastoma both in vitro and in vivo, with promising results for specifically two novel compounds.

## 2. Materials and Methods

### 2.1. Synthesis of the Compounds

The synthesis of FRB21, FRB24, FRB44 and FRB56 was performed following a previous description [9]. Briefly, TLC using precoated silica gel aluminum plates containing a fluorescent indicator was used to monitor reactions (Merck, Darmstadt, Germany, 5539). Detection was performed by UV (254 nm) followed by charring with sulfuric–acetic acid spray, 1% aqueous potassium permanganate solution or 0.5% phosphomolybdic acid in 95% EtOH. Anhydrous Na_2_SO_4_ was used to dry organic solutions during work-ups and the removal of solvents was carried out under vacuum with a rotary evaporator. Flash column chromatography was performed using silica gel 60 (230–400 mesh, Merck). Melting points were determined on a Kofler block and are uncorrected. The 1H NMR spectra were recorded with a Varian VXR-200S spectrometer (Varian, Inc., Palo Alto, CA, USA), using tetramethylsilane as an internal standard, and 13C NMR spectra were recorded with a Bruker WP-200-SY (Bruker Corp., Billerica, MA, USA). All the assignments for protons and carbons were in agreement with 2D COSY, HSQC, HMBC and 1D NOESY.

### 2.2. Cell Lines and Cultures

U87-MG, U251-MG and NCI-H1299 cells were purchased from ATCC, while MKN45 was obtained from the Leibniz Institute DSMZ-German Collection of Microorganisms and Cell Cultures and the RWP1 cell line was kindly provided by Dr. Francisco Real. Cells were cultured in DMEM or RPMI (Gibco, Waltham, MA, USA) supplemented with 10% FBS, 100 U/mL penicillin and streptomycin and 2 mM L-Glutamine. Patient-derived glioma neural stem cells (GNS166 and GNS179) were kindly provided by Dr. Steve Pollard. These cells were cultured in adhesion plates treated with laminin A in DMEM/F12 media (Gibco) supplemented with N2 (Gibco), B27 (Gibco), D-(+)-Glucose solution 45% (Sigma-Aldrich, Burlington, MA, USA), basic Fibroblast Growth Factor (Gibco) and epidermal growth factor (Sigma-Aldrich). Normal human astrocytes (NHAs) were purchased from ScienCell and cultured using an astrocyte medium kit (ScienCell, San Diego, CA, USA) supplemented with FBS and Astrocyte Growth Supplement (AGS). All cells were maintained at standard conditions of 37 °C, 95% humidity, 21% O_2_ and 5% CO_2_, and cultures were regularly tested for Mycoplasma.

### 2.3. Online Prediction Tools

Physicochemical properties of the compounds being studied as well as the number of exerted violations were predicted by introducing their structure into Molinspiration (https://www.molinspiration.com/cgi/properties, accessed on 30 November 2024). Further, Online BBB Predictor (http://ssbio.cau.ac.kr/software/logbb_pred/, accessed on 30 November 2024) was used to predict the ability of the compounds to cross the BBB. For this, the parameters established by default in the tool were maintained, and prediction was performed by introducing the structure of the compounds being studied. Finally, the potential toxicity risks of the compounds were assessed with OSIRIS Property Explorer (https://www.organic-chemistry.org/prog/peo/, accessed on 30 November 2024).

### 2.4. Study of Cell Viability

Cells were seeded in 96-well plates at a density of 1.5 × 10^3^ or 5 × 10^3^ cells/well (conventional cell lines or GNS and NHAs, respectively) and incubated overnight. Next, cells were treated with increasing concentrations of the compounds, and after 72 h, they were incubated for 3.5 h with MTT. Then, absorbance at 570 nm was measured. GraphPad Prism software version 10.4.1 was used to calculate the IC_50_ values.

### 2.5. Western Blot

Sodium dodecyl sulfate–polyacrylamide gel electrophoresis was performed. Primary antibodies against HDAC1 (Abcam, Cambridge, UK) and HDAC6 (Cell Signaling, Dancers, MA, USA) and their main targets, acetylated H3 (Cell Signaling) and acetylated α-tubulin (Abcam), were used; acetylated lysine (Cell Signaling) and β-actin (Sigma-Aldrich) were used as endogenous controls. Secondary antibodies conjugated to horseradish peroxidase were anti-rabbit or anti-mouse (Cell Signaling). Proteins were detected using the iBright imaging system and NOVEX ECL Chemi Substrate or SuperSignal West Femto Maximum Sensitivity Substrate (ThermoFisher, Waltham, MA, USA).

### 2.6. Enzyme Inhibition Assays

For MAO inhibition studies, the enzyme solution (0.5 U/mL recombinant hMAO-A or 1.5 U/mL recombinant hMAO-B) was incubated with an inhibitor solution containing 10 µM of the compounds for 30 min at 37 °C. Next, horseradish peroxidase (200 U/mL), Ampliflu Red (20 mM) and tyramine hydrochloride (100 mM) (Sigma-Aldrich) were added, and absorbance was measured at 570 nm every 5 min for 30 min. The percentage of inhibition was calculated from the absorbance values. For the compounds exhibiting greater than 50% inhibition, IC_50_ values were determined by repeating the assay using increasing concentrations of the inhibitor.

The inhibitory capacity of the compounds on ChEs was assessed using Ellman’s colorimetric method. Briefly, an inhibitor solution containing 10 µM of the MSMs and 0.3 mM DTNB was incubated for 20 min with the enzyme solution (0.5 U/mL recombinant hMAO-A or 0.25 U/mL recombinant hMAO-B) at 37 °C. After the incubation, the substrate solution, either acetylthiocholine iodide or butyrylthiocholine iodide (Sigma-Aldrich) at 1.5 mM, was added, and absorbance was measured at 405 nm every minute for 10 min. Inhibition percentages were calculated from these values. For compounds showing more than 50% inhibition, IC50 values were determined by repeating the assay with increasing concentrations of the inhibitor. Further, enzyme kinetics were studied using different substrate and inhibitor concentrations. Results were represented in a Lineweaver–Burk plot using GraphPad Prism, and the constant of inhibition (Ki) was estimated from the point at which the line cuts the *x*-axis in the secondary plot.

### 2.7. Immunofluorescence

For immunofluorescence assays, cells were seeded at a density of 2 × 10^4^ cells/well in 8-well immunofluorescence chambers (LabTek Thermo, Waltham, MA, USA) and incubated overnight. Then, cells were treated for 48 h and fixed with 4% PFA. Primary antibodies against phospho-histone H3 (p-H3) (Abcam), cleaved caspase-3 (Casp3) (R&D Systems, Minneapolis, MN, USA) and Ki67 (Abcam) and secondary antibodies Alexa Fluor anti-mouse and anti-rabbit (Invitrogen, Carlsbad, CA, USA) were used. Nuclei were stained with DAPI (Sigma-Aldrich). Pictures were taken with an Axio Observer 7 epifluorescence microscope, and Qupath version 0.6.0 was used for quantifications. Percentages of Ki67-, p-H3- and Casp3-positive cells were calculated, taking into account the number of nuclei.

### 2.8. Senescence-Associated β-Galactosidase

Cells were incubated and treated following the same procedure as described for the immunofluorescence section. The senescence assay was performed using the Senescence β-Galactosidase Staining Kit (Cell Signaling) according to the manufacturer’s guidelines.

### 2.9. Cell Cycle

Treated cells were harvested using accutase and washed with PBS. For fixation, 1 × 10^6^ cells were treated with 70% cold ethanol (−20 °C) for a minimum of 24 h. Permeabilization was performed by incubating the cells with 0.5% Triton X-100 and 25 µg/mL RNase A in PBS for 30 min at room temperature. DNA was then stained for 15 min with 25 ng/mL propidium iodide and samples were analyzed using a CytoFLEX flow cytometer (Beckman Coulter Co., Brea, CA, USA).

### 2.10. RNA-Seq and Proteomic Studies

RNA-seq and proteomic studies were performed for 5 µM of FRB44 and FRB56 in GNS166. For transcriptomic analysis, total RNA was extracted and samples were sent to BGI Tech Solutions. On average, 44 million clean reads were generated per sample, and DESeq2 package (version 1.44.0) was used for differential expression analysis using default settings. Genes presenting a fold-change value ≥4 or ≤−4 and an FDR-adjusted *p*-value < 0.01 were defined as differentially expressed and used to perform downstream enrichment studies.

Proteomic studies were performed using label-free relative quantification on nLC MS/MS in the Proteomic Facility of CIC bioGUNE. Overrepresentation analysis (ORA) of significantly differentially expressed proteins (*p* < 0.05) was performed with R package clusterProfiler version 4.16.0. The mass spectrometry proteomic data have been deposited to the ProteomeXchange Consortium via the PRIDE partner repository with the dataset identifier PXD069713.

For both RNA-seq and proteomics, the top 20 enriched terms were plotted. We then performed a combined study of both datasets. This involved first conducting independent enrichment analysis on the RNA-seq and proteome data, and stratifying by upregulated and downregulated entities across all three Gene Ontology domains, using all genes as the background. Following this, the top 25 enriched gene sets were clustered based on the Jaccard distances. Additionally, genes and proteins that were significantly differentially expressed in both approaches for each cluster were selected, and Spearman correlation analyses were performed with the target genes of the compound being studied using data from the TCGA cohort.

### 2.11. Enrichment Analysis of Senescence

Senescence signatures and associated pathways obtained from the Molecular Signature Database (https://www.gsea-msigdb.org/gsea/msigdb, accessed on 17 November 2025) were analyzed using RNA-seq and proteomic FRB studies by gene set enrichment analysis (GSEA).

### 2.12. Cell Type Calling from Bulk Proteomic Data

In addition, human cell type markers were downloaded from the CellMarker database [24]. These were used to perform ORA on the proteomic data to assess which cell types could be targeted upon FRB44 or FRB56 treatment.

### 2.13. Single-Cell Transcriptomic Analysis of Common FRB44 and FRB56 Target Signatures

Publicly available single-cell RNA sequencing (scRNA-seq) data from the GBM Space resource [25,26] (https://www.gbmspace.org/, accessed on 17 November 2025) were acquired to analyze the transcriptional expression of the pharmacological targets for compounds FRB44 and FRB56. Predefined cellular states established by the original authors were utilized for this analysis. The expression data were normalized according to the standard Scanpy processing pipeline. For each of the common clusters obtained in the FRB44 and FRB56 omics (i.e., cell cycle, synapse, transport and vesicle clusters), a composite gene expression score was created within this scRNA-seq dataset. These were generated using the genes commonly perturbed by the two compounds, as identified in both RNA-seq and proteomic analyses.

### 2.14. RNA Extraction and RT-qPCR

Total RNA was extracted using Trizol (Life Technologies, Carlsbad, CA, USA). Then, reverse transcription (RT) was performed with the Maxima First Strand cDNA Synthesis Kit (ThermoFisher) following the manufacturer’s guidelines. Quantitative PCR (qPCR) was carried out in a CFX384 Thermal Cycler (BioRad, Hercules, CA, USA) using Absolute SYBR Green mix (ThermoFisher). In order to correct variations in initial cDNA levels, 18S was used as a housekeeping gene. The 2^−ΔΔCt^ formula was used to determine the relative gene expression.

### 2.15. In Vivo Experiments

First, 50 mg/mL of FRB44, FRB56 or vehicle (DMSO) was intraperitoneally injected into athymic nude-Foxn1^nu^ mice following a schedule of 3 days per week, and changes in body weight were measured to assess the potential toxicity of the compounds. At the end-point, macroscopic analysis of the different organs was performed.

For carcinogenesis studies, subcutaneous tumor growth experiments were performed via the injection of 5 × 10^5^ cells into two bottom flanks of athymic nude-Foxn1^nu^ mice. Once tumors reached a size of 25–50 mm^3^, mice were sorted into different treatment groups based on their gender and tumor size. An external caliper was used for tumor size measurement, and the tumor volume was estimated using the formula V  =  L·W^2^·0.5 (where L is tumor length and W is tumor width). Mice were intraperitoneally treated with vehicle or 50 mg/kg of FRB44 or FRB56 following a schedule of 3 days per week for 4 weeks. Sample size was calculated, taking into account that control groups could have an average size of 700 mm^3^ and we wanted to detect a difference of ~300 mm^2^. For the cytotoxic experiment, 8 controls and 8 treated animals were used, while for the efficacy experiment 12 control animals and 8 treated animals were used.

### 2.16. Immunohistochemistry

Subcutaneous tumors were extracted, fixed with 4% formaldehyde for 48 h and embedded in paraffin. Tumor sections were obtained and stainings for Ki67 (Ab16667, Abcam), acetyl H3 (9649, Cell Signaling) and acetylated α-tubulin (ab179484, Abcam) were performed. A ZEISS Axioscan 7 slide scanner was used to capture images of the stainings, and analyses were performed using Qupath.

### 2.17. Statistical Analysis

Statistical analyses were performed using GraphPad Prism software (version 8.3.0, San Diego, CA, USA). Data were represented as mean values ± standard error of the mean (SEM). Differences between three or more groups were generally assessed using a one-way ANOVA test, comparing each experimental column against the mean of the control column. The Dunnett test was applied as the post hoc correction method for these comparisons. For in vivo tumor growth experiments, a two-way ANOVA test was conducted, using the mixed-effects model method. A threshold of *p* < 0.05 was adopted to define statistical significance (*p* ≤  0.1 #, *p* ≤ 0.05 *, *p* ≤ 0.01 ** and *p* ≤ 0.001 ***).

### 2.18. Ethics Approval

This study was approved by the Clinical Research Ethics Committee of the Basque Country (CEIm-E, protocol PI2023104) and adheres to the Declaration of Helsinki. All animal procedures were approved by the Research Animal Care committees of the Biogipuzkoa Institute and Gipuzkoa County (PRO-AE-SS-331, 30 September 2024).

## 3. Results

### 3.1. Novel MSMs for the Treatment of GBM: Physicochemical Properties and Cytotoxicity

**Figure 1:**
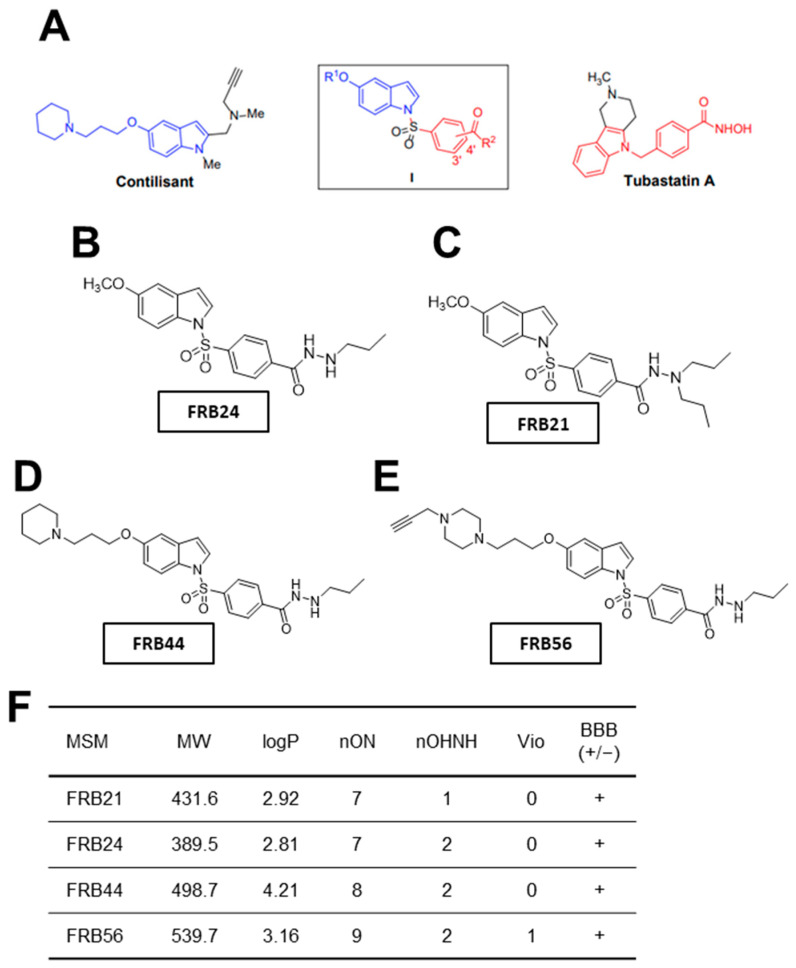
**Novel FRB compounds inhibit target genes.** (**A**) Schematic representation of the Contilisant (in blue) and Tubastatin A (in red) chemical structures. Schematic representation of the chemical structures of (**B**) FRB24, (**C**) FBR21, (**D**) FRB44 and (**E**) FRB56. (**F**) Table summarizing the physicochemical properties of the four MSMs and their predicted ability to cross the BBB.

In this work we have specifically characterized four different multitarget small molecules (FRB21, FRB24, FRB44 and FRB56). In Figure 1A we can see a schematic representation of the structural origin of the novel compounds, which are polyfunctionalized indole derivatives designed by combining selected pharmacophoric groups from reference ligands Contilisant and Tubastatin A. Figure 1B–E show the final structures of the tested compounds. Moreover, the structures of FRB21, FRB24, FRB44 and FBR56 have been confirmed by 1H NMR and 13C NMR spectral data (Figure S1A–H).

Once the compounds were synthesized, physicochemical properties of the FRBs were predicted using Molinspiration. The number of violations gives an idea of how drug-like the structure of a compound is. Thus, the fewer violations a compound displays, the more drug-like its properties are [27]. All compounds presented no violations, except FRB56, which had one violation because its molecular weight slightly exceeds the 500 g/mol limit defined by Lipinski’s rule (Figure 1F). Because one major challenge in developing brain-targeted drugs is crossing the blood–brain barrier (BBB), we used the online CBLigand BBB predictor to estimate the compounds’ BBB permeability from their structures [28]. According to this predictor, all FRB compounds are likely to cross the BBB (Figure 1F). Finally, we assessed toxicity risks using the OSIRIS Property Explorer. Specifically, we evaluated the mutagenic, tumorigenic and irritant potential of the FRBs. Importantly, none of the compounds appeared to be mutagenic, tumorigenic or irritant.

**Figure 2:**
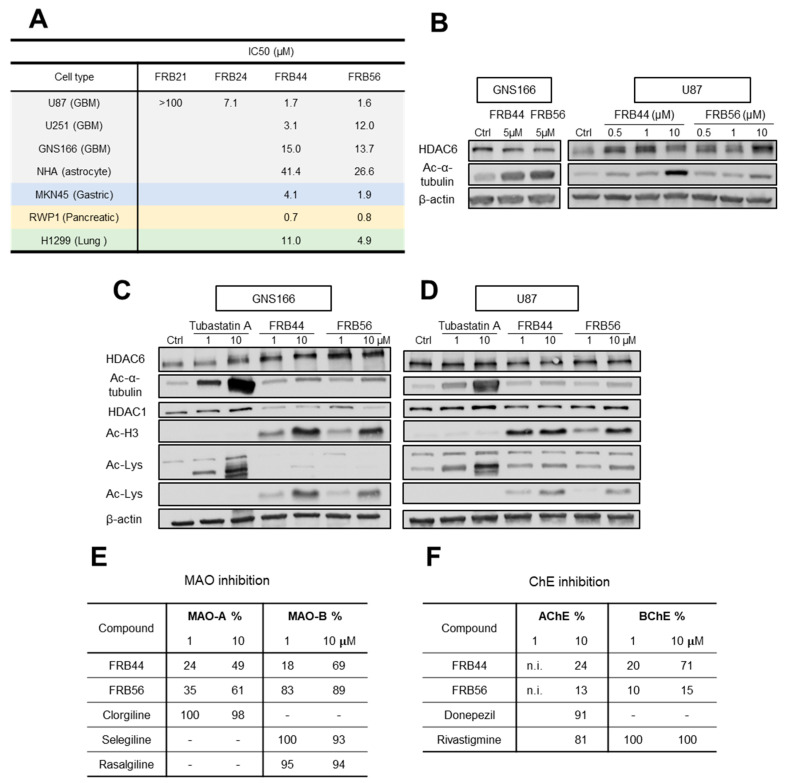
**Novel FRB compounds present antitumor activity.** (**A**) IC_50_ values of FBR21, FRB24, FRB44 and FRB56 in the glioblastoma (U87), glioma (U251), patient-derived GSC (GNS166), primary human astrocyte (NHA), gastric cancer (MKN45), pancreatic cancer (RWP1) and lung cancer (H1299) cell lines measured by MTT. (**B**) WB of HDAC6 and its target (acetylated α-tubulin) in GNS166-treated cells and U87-treated cells. (**C**) HDAC6, acetylated α-tubulin, HDAC1, acetylated histone 3 and total acetylated lysine protein expression in FRB- and Tubastatin A-treated GNS166 cells and (**D**) U87 cells. (**E**) MAO-A and MAO-B inhibitory capacity of FRBs and reference compounds Clorgiline, Selegiline and Rasalgiline. (**F**) AChE and BChE inhibitory capacity of FRBs and reference compounds Donepezil and Rivastigmine. n.i.: No inhibition.

We and others have observed in different datasets that HDACs, MAOs and ChE (primarily MAO-B and BChE) are expressed across different cell types within tumors, being high in malignant and progenitor-like cells (Figure S2A–D) [9]. The high expression of our targets in the most malignant cells strongly suggests that their inhibition represents a promising therapeutic strategy for glioblastoma. Thus, we performed cytotoxicity screening of the four compounds by MTT assays in U87 glioma cells. Three out of four MSMs presented IC_50_ values lower than 10 μM, with FRB44 and FRB56 showing IC_50_ values of ~1.5 μM (Figure 2A).

Consequently, we selected these two molecules and completed MTT assays in additional glioma cells. Thus, we detected similar IC_50_ values in U251 glioma cells and in patient-derived glioma neural stem cells (GNS166) (Figure 2A). In parallel, MTT assays were also performed in primary human astrocytes (NHAs) to assess toxicity in non-tumor cells. In both FRB44 and FRB56 compounds, the IC_50_ values measured in the cancer cells were consistently lower than those observed in non-tumor cells, suggesting that both compounds may be safe for healthy cells while exerting cytotoxic effects in cancer cells (Figure 2A). To further support the potential antitumor activity of the selected compounds, we evaluated their cytotoxicity in gastric (MKN45), pancreatic (RWP1) and lung (H1299) cancer cell lines, where they showed potential antitumor effects, especially the FRB56 compound, with IC_50_ values lower than 5 μM in all cell lines (Figure 2A).

### 3.2. Target Inhibition of FRB MSM Molecules

We then evaluated the effect of FBR44 and FRB56 on their molecular targets, specifically assessing inhibitory activity on HDAC6, MAOs and ChEs. To determine whether the selected compounds inhibited HDAC6 activity, we checked the expression of its downstream target (acetyl-α-tubulin) as well as total HDAC6 in GNS166 and U87 cells. Both compounds increased the levels of acetyl-α-tubulin in both glioma cell types in a dose-dependent manner, confirming HDAC6 inhibition (Figure 2B). We next compared the effect of the novel compounds with Tubastatin A and checked that this molecule produced higher levels of acetylated α-tubulin than the FRBs in glioma cells (Figure 2C,D). We next evaluated the inhibitory effect on other HDACs such as HDAC1 as well as total lysine acetylation of proteins. We detected that both FRBs increased the expression of acetylated histone 3, the target of HDAC1, as well as total lysine acetylation (Figure 2C,D).

After checking HDAC inhibition, we assessed MAOs using MAO inhibition assays that mimic the enzymatic reaction in cells. As an initial screening, we measured percentage inhibition of MAO-A and MAO-B for each compound at 1 and 10 µM. This screening showed that FRBs inhibited both MAO isoforms in a dose-dependent manner, with stronger inhibition of MAO-B than MAO-A, reaching 70 and 90% of inhibition with 10 µM of FRB44 and FRB56, respectively (Figure 2E). Thus, both compounds were able to inhibit MAO-A, and especially MAO-B. The comparison with specific MAO inhibitors such as clorgiline (specific MAO-A inhibitor) and selegiline or rasalgiline (MAO-B inhibitors) showed similar rates for the effect of FRBs, especially in the case of FRB56 and MAO-B inhibition. Following this screening, we conducted additional MAO inhibition assays using increasing concentrations of each FRB. IC_50_ values were calculated from the representation of change in absorbance against the negative logarithm of inhibitor concentration (Figure S3A). For the case of MAO-A, FRB56 was the only FRB to be tested, and presented an IC_50_ value of 1.68 µM. For MAO-B, IC_50_ values for FRB44, FRB56 and reference compounds selegiline and rasalgiline were calculated, which were in the range between 0.15 µM and ~3 µM.

We next evaluated ChE activity using a similar approach. First, percentage inhibition of AChE and BChE was measured at 1 and 10 µM using Ellman’s colorimetric method (Figure 2F). In the case of the AChE inhibition study, the FRBs exerted inhibition that ranged between 13 and 24% at 10 µM, lower than the reference inhibitor for AChE, donepezil, and the ChE inhibitor, rivastigmine (Figure 2F). The inhibition effect of FRBs was higher in BChEs, with FRB44 reaching 70% at 10 µM. Consequently, the IC_50_ of BChE was only calculated for this compound by following the same protocol as for the calculation of inhibition percentage, but using increasing concentrations of the compound (Figure S3B). Finally, we characterized the inhibition mechanism and inhibition constant (K_i_) for FRB44 on BChE (Figure S3C,D). Using multiple substrate and inhibitor concentrations, we measured absorbance over time and plotted the results as double-reciprocal (Lineweaver–Burk) plots to infer inhibition type. FRB44 exhibited mixed inhibition, as lines corresponding to different FRB44 concentrations intersected in the first quadrant (Figure S3C), indicating reversible binding to both the free enzyme and the enzyme–substrate complex with differing affinities. Slopes from the Lineweaver–Burk plots were then plotted in a secondary plot to determine K_i_, calculated from the negative *x*-axis intercept (Figure S3D).

### 3.3. FRB44 and FRB56 Reduce Cell Proliferation and Promote Apoptosis

Next, we evaluated the potential antitumor effect of FRBs, studying cell proliferation and apoptosis in patient-derived GSCs and in glioma cells, and compared them to Tubastatin A. For this, cells were cultured with increasing doses for 48 h, and the expression of different markers, Ki67 and phospho-histone 3 (p-H3) for proliferation and cleaved caspase-3 for apoptosis, was analyzed.

**Figure 3:**
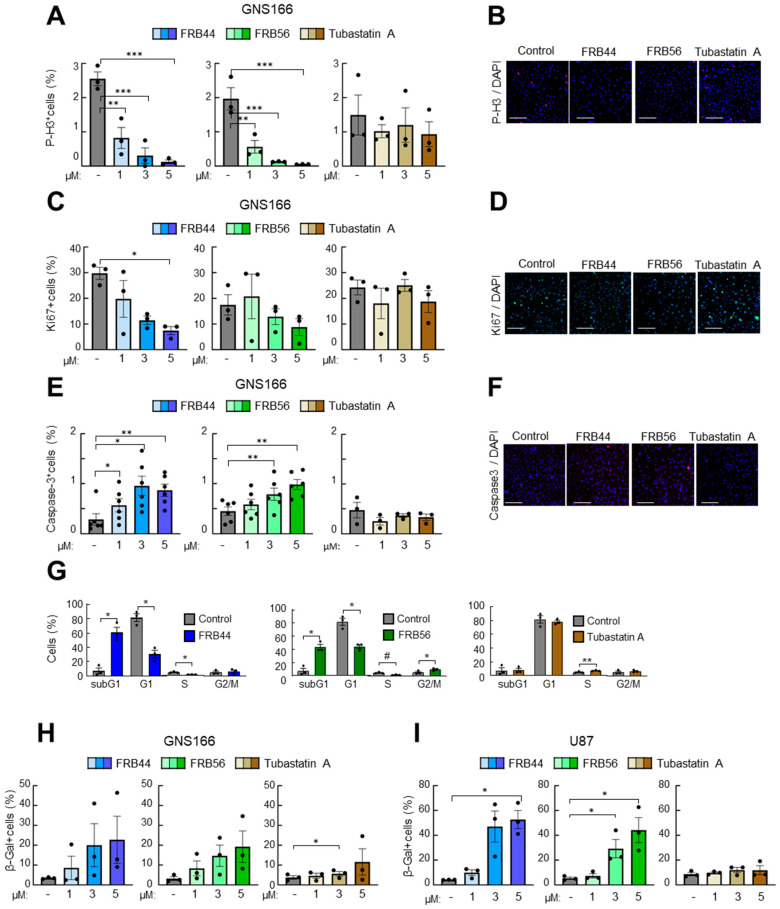
**FRB44 and FRB56 are cytotoxic for patient-derived GSCs.** (**A**,**B**) Quantification and representative pictures of p-H3-positive, (**C**,**D**) Ki67-positive and (**E**,**F**) cleaved caspase-3 cells after 1, 3 and 5 µM of FRB44, FRB56 and Tubastatin A treatments in GNS166 cells (*n* ≥ 3). Scale bar 200 μM; immunofluorescence images are included after 5 μM of treatment. (**G**) Cell cycle study in GNS166 cells after administration of 10 µM of FRB44, FRB56 and Tubastatin A (*n* ≥ 3). (**H**,**I**) β-galactosidase quantification of GNS166 and U87 cells treated with 1, 3 and 5 µM of FRB44, FRB56 and Tubastatin A. *p* ≤  0.1 #, *p* ≤ 0.05 *, *p* ≤ 0.01 ** and *p* ≤ 0.001 ***.

We started with treating both FBR compounds and Tubastatin A in two patient-derived glioma stem cells (GNS166 and GNS179 cells) as well as two glioma cell lines (U87 and U251), using 1, 3 and 5 μM concentrations. We detected clear reductions in both proliferative markers in all glioma cell types after FRB44 and FRB56 treatments, in a dose-dependent manner (Figure 3A–D and Figure S4A–F). The effect was significantly higher than with the administration of the same concentrations of Tubastatin A. We also found that both FRB compounds increased the number of positive cells for caspase-3 in a dose-dependent manner in the four glioma cells, revealing the accumulation of cell apoptosis (Figure 3E,F and Figure S4G–J). On the contrary, Tubastatin A slightly increased cleaved caspase-3-positive cells, but these differences were not statistically significant (Figure S4G). In order to confirm the effects on proliferation and apoptosis, we assessed cell cycle distribution by flow cytometry. Treatment with the FRB compounds induced a marked increase in the sub-G1 population, associated with apoptotic cell death, and a reduction in the G1 and S phases in glioma cells. These findings were much more intense and significant than in the case of Tubastatin A-treated glioma cells (Figure 3G).

Finally, we checked the effect of the compounds on cellular senescence in GNS166 and U87 cells, measured by Senescence-Associated Beta Galactosidase-positive cells, and found that both FRBs increased cell senescence in glioma cells at a higher rate than Tubastatin A (Figure 3H,I).

### 3.4. FRBs Alter Cell Cycle in Both RNA-Seq and Proteomics in GSCs

To identify the genes whose expression is modulated by FRBs and to elucidate the molecular mechanisms underlying the activity of FRB44 and FRB56, we performed RNA-seq and proteomic analyses on GNS166 cultured in the absence or presence of 5 µM of the two compounds independently.

**Figure 4:**
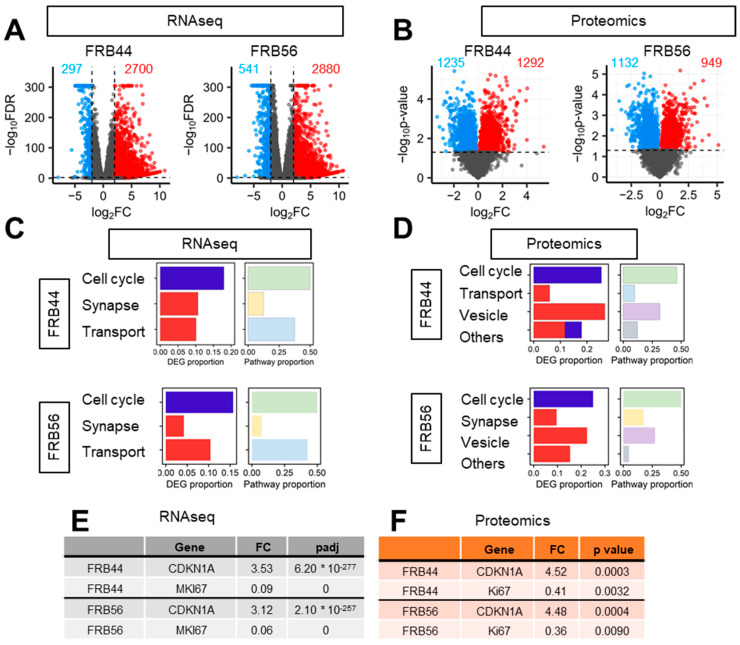
**Both FRB44 and FRB56 treatments deregulate the cell cycle in GSCs.** (**A**,**B**) Volcano plot of RNA-seq and proteomics based on a fold change > 4 and *p*-value < 0.01. (**C**) Proportion of DEGs and biological processes represented by the biological themes in RNA-seq in FRB44- and FRB56-treated cells. (**D**) Proportion of DEPs and biological processes represented by the biological themes in proteomics in FRB44- and FRB56-treated cells. (**E**) Fold changes and adjusted *p*-values of *CDKN1A* and *MKI67* in RNA-seq studies. (**F**) Fold changes and *p*-values of *CDKN1A* and *MKI67* in proteomic studies.

Principal component analysis of RNA-seq studies (PCA) clearly separated the expression profiles of untreated cells from those treated with FRB44 and FRB56 (Figure S5A). These differences were further confirmed by volcano plots, using thresholds of fold changes ≥ 5 and *p*-values < 0.01, which revealed 2700 and 2880 upregulated genes in FRB44- and FRB56-treated cells, respectively, and 297 and 541 downregulated genes (Figure 4A). Gene Ontology (GO) analyses were also performed on RNA-seq data in both FRB44- and FRB56-treated cells, and in both cases, they indicated downregulation of pathways related to cell cycle, while pathways related to synapse and transport/channel activity were upregulated (Figure 4C). Among the cell cycle genes, the alteration in Ki67 and CDKN1A (p21^CIP^) expression is noticeable, as they have 0.09/0.06- and 3.53/3.12-fold changes in cells treated with FRB44 and FRB56, respectively (Figure 4E). Moreover, processes related to mitosis and chromosome segregation are downregulated, which could indicate that cells have entered a phase of growth arrest which could lead to a later apoptosis or senescence process (Figure S6A,B). Indeed, senescence-related processes and pathways were significantly altered in cells treated with FRBs. The enrichment of pathways related to the synaptic and postsynaptic membranes, among others, is clustered in the synapse group. Moreover, we see an upregulation of genes related to ion channels and transporters (clustered in the transport group), especially those that are voltage-gated, which matches well with the synapse category. Figure 4C shows the proportions of DEGs and pathways represented by these biological themes (cell cycle, synapse and transport activity) in both FRB44 and FRB56 treatments, showing the three groups already mentioned.

We followed the same approach for the proteomic studies in GNS166 cells treated with FRB44 or FRB56. As happened with the RNA-seq studies, PCA plots from FRB44 and FRB56 clearly separate the expression profiles of control and treated cells (Supplementary Figure S5B). Volcano plots using thresholds of fold changes ≥ 5 and *p*-values < 0.05 show 1292 and 949 differentially upregulated proteins and 1235 and 1132 downregulated proteins for FBR44- and FRB56-treated cells compared to controls, respectively (Figure 4B). Likewise, proteomic GO analysis indicated downregulation of cell cycle pathways (mostly related to mitosis and DNA repair), while pathways related to vesicles (lysosomes and vacuoles) and ion homeostasis, among others, were upregulated after FRB44 treatment (Supplementary Figure S7A). In FRB56-treated cells, GO analysis indicates downregulation of cell cycle pathways, whereas pathways associated with synaptic function and vesicle biology (including increases in secretion, ion channels and membrane traffic), among others, are significantly upregulated (Supplementary Figure S7B). Indeed, as is presented in Figure 4D, the cell cycle- and vesicle-related pathways are the ones shared between both treatments. As in the case of RNA-seq, the levels of Ki67 and CDKN1A (p21^CIP^) were markedly reduced and increased, respectively (Figure 4F), as was the case for senescence-related pathways (Figure S8).

**Figure 5:**
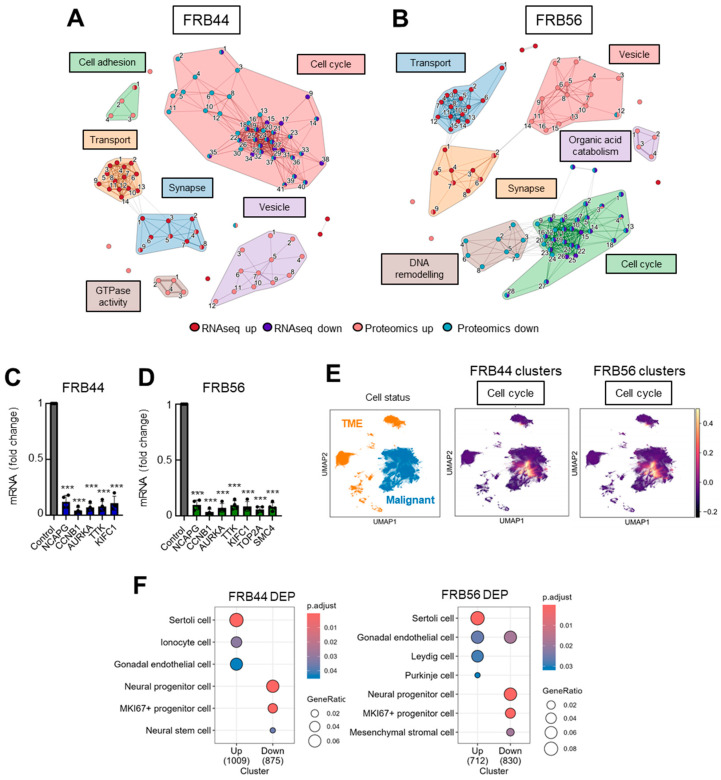
**The cell cycle is a common biological theme after multiomic integration.** (**A**) Clustering of top 25 up- and down-enriched pathways in a combined analysis of RNA-seq and proteomics for FRB44 and (**B**) FRB56. The width of the connecting bars is indicative of the number of genes that are shared between the two pathways. The full list of pathways is available in Figure S8A,B. (**C**) q-RT-PCR of selected DEGs from the cell cycle cluster in GNS166 non-treated and FRB44- and (**D**) FRB56-treated cells (n = 4). (**E**) Uniform Manifold Approximation and Projection (UMAP) visualization displaying the predefined cellular states from the original study, segmented into malignant cells and the tumor microenvironment (TME), and UMAP visualization of the GBMspace dataset depicting the mean module scores derived from the commonly differentially expressed genes (DEGs) within the cell cycle cluster defined by the FRB44 and FRB56 multiomic analyses. (**F**) Human cell type marker ORA from proteomic studies, based on data from the CellMarker database. *p* ≤ 0.001 ***.

We complemented the GO results with an integrated analysis of RNA-seq and proteomic data in both treatments. The top 25 enriched upregulated and downregulated pathways were selected and clustered by Jaccard similarity, yielding six distinct clusters (Figure 5A,B). Notably, cell cycle- and synapse-related pathways were shared between RNA-seq and proteomic datasets in both FRB44- (Figure 5A) and FRB56 (Figure 5B) -treated cells. All the processes are shown in Supplementary Figure S9. Given its prominence, the cell cycle-related cluster was selected for further characterization. To this end, we performed a correlation analysis between the genes altered at both the RNA and protein levels and the targets of FRBs previously checked (HDAC1, HDAC6, MAO-A, MAO-B, ACHE and BCHE) (Supplementary Figure S10). Indeed, the expression of genes involved in the cell cycle showed a significant positive correlation with HDACs and BChE with patients from the TCGA cohort. Moreover, we validated some of these genes by RT-q-PCR in FRB44- and FRB56-treated GNS166 cells, and demonstrated that the expression of these genes was also decreased after both treatments (Figure 5C,D).

Next, we took advantage of publicly available datasets in order to further characterize and understand the molecular mechanisms underlying FRB treatment. First, using publicly available single-cell RNA sequencing (scRNA-seq) data from the GBM Space resource (https://www.gbmspace.org/, accessed on 17 November 2025), we identified that the differentially expressed cell cycle cluster is specifically associated with malignant and proliferative cells. In contrast, the synapse clusters were related to neurons (Figure 5E and Figure S11). Additionally, using the CellMarker database we identified that the proteins decreased after the treatment with FRB44 and FRB56 are described markers of Ki67 progenitor cells and neural stem or progenitor cells (Figure 5F).

### 3.5. Both FRB44 and FRB56 Reduce Tumor Growth In Vivo

**Figure 6:**
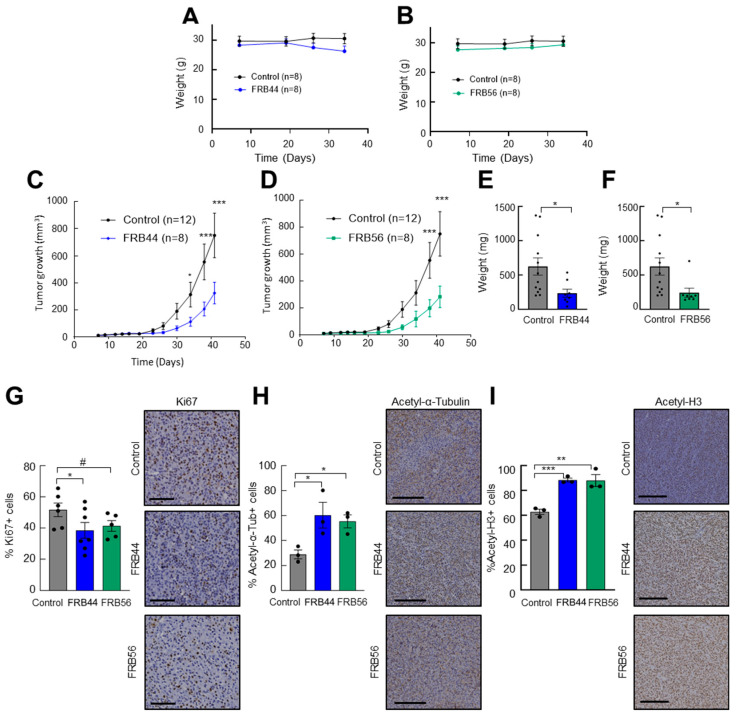
**FRBs reduce tumor growth in vivo.** (**A**) Weights of control and FRB44- and (**B**) FRB56-treated mice. (**C**) Measurements of tumor volume at indicated time points of mice treated intraperitoneally with vehicle (control) or 50 mg/kg of FRB44 and (**D**) FBR56 compounds. (**E**) Tumor weights at the final point of the experiment in control and FRB44- and (**F**) control and FRB56-treated animals. (**G**) Percents of Ki67-positive cells assessed by IHC in control and FRB44- and FRB56-treated animals. (**H**) Percents of acetylated α-tubulin-positive cells and (**I**) acetylated histone-3-positive cells assessed by IHC (n ≥ 3). The scale bar is 200 μM. *p* ≤  0.1 #, *p* ≤ 0.05 *, *p* ≤ 0.01 ** and *p* ≤ 0.001 ***.

Taking into account the promising in vitro and OMIC results for FRB44 and FRB56, we next examined their in vivo effects on subcutaneous tumor growth. First, we evaluated the toxicity of the compounds by checking the body weight of the treated animals. As shown in Figure 6A,B, there were no statistically significant differences in body weight between control animals and those treated with FRB44 or FRB56. In addition, even though these were not quantified, no clear differences were observed in the macroscopic analysis between the different organs of the animals among the experimental groups at the end-point.

Next, we studied their antitumor effects. For this, U87 cells were subcutaneously injected into mice, and once tumors reached 25–50 mm^3^, animals were randomly assigned and treated intraperitoneally with vehicle or 50 mg/kg of FRB44 or FRB56. Treatment with either FRB44 or FRB56 produced a significant reduction in tumor growth compared with controls: control tumors reached a final volume of ~750 mm^3^, whereas tumors from FRB44- and FRB56-treated mice reached ~325 mm^3^ and ~280 mm^3^, respectively (Figure 6C,D). These differences were also reflected in final tumor weights, with mean weights in the FRB44 and FRB56 groups being 62.5% and 61.4% lower compared to controls, respectively (Figure 6E,F). To further corroborate the antitumor effect and to investigate whether in vitro and OMIC data were recapitulated in vivo, we performed immunohistochemistry staining for the proliferation marker Ki67 in resected tumors. As shown in Figure 6G, FRB44 and FRB56 promoted a decrease in the number of Ki67-positive cells of around 30%. Finally, we also wanted to confirm whether the targets of FRBs were affected, as well as the antitumor effect related to their inhibition. For this, we completed immunohistochemistry staining for acetyl-α-tubulin, the target of HDAC6, and acetyl histone 3, the target of HDAC1. These analyses revealed increases in the expression of both acetyl-α-tubulin and acetyl histone 3 (Figure 6H,I) confirming that the FRBs inhibit HDACs in vivo.

## 4. Discussion

Multifactorial diseases such as cancer are characterized by complex molecular alterations and high intra- and inter-tumor heterogeneity, which limit the effectiveness of single-target therapies and contribute to recurrence and therapy resistance. This is particularly evident in glioblastoma, one of the most aggressive brain tumors, where the presence of GSCs, infiltrative growth and molecular diversity result in poor patient prognosis and highlight the urgent need for novel therapeutic strategies.

In this context, multitarget small molecules are emerging as a promising approach to circumvent the limitations of conventional therapies. In this work, we describe two novel compounds, FRB44 and FRB56, which demonstrated promising multitarget activity with significant potential for GBM therapy. Our integrated in vitro, ex vivo and in vivo analyses confirm that these compounds possess both favorable drug-like properties and potent antitumor activity. Mechanistically, FRB44 and FRB56 exhibited inhibitory activity against HDAC6, MAO-B and BChE. As the expression of H3R and S1R is also reported to be increased in glioblastoma and in other types of cancer [29,30,31], we cannot exclude the possibility that some of the results obtained in this work are mediated by the modulation of H3R and S1R. In this line, previous studies have described the effect of MSMs against some of the targets described in this study. Thus, a novel dual inhibitor of MAO-A and HDACs presented the capacity to inhibit cell growth and promote cell death [32]. On the other hand, dual modulators exerting S1R agonism and HDAC inhibition presented antitumor effects on glioblastoma, inhibiting cell growth and inducing cell death of both TMZ-sensitive and -resistant cells [33]. In our case, we went a step further and generated pentatarget molecules. Thus, by simultaneously modulating several relevant pathways, the novel FRBs may effectively target both proliferative tumor cells and the quiescent GSC population responsible for tumor relapse. Indeed, HDAC6 has been associated with glioma stem cell survival, and inhibition of HDAC6 has been linked to GSC differentiation and increased sensitivity to chemotherapy [34,35]. Moreover, MAOs have been identified as highly expressed in gliomas and correlated with tumor grade [36], and it has been reported that the activity of ChEs is increased in brain tumors, where cholinergic signaling influences tumor progression [37]. Indeed, we further confirmed that HDAC6 and MAO-B and BChE are predominantly expressed in malignant and progenitor cells.

FRBs were generated based on Tubastatin A, which has been broadly considered as a specific HDAC6 inhibitor; it is also able to inhibit HDAC10, and exerts some effect on other HDACs including HDAC1 [38,39]. We confirmed that FRB compounds also present higher HDAC1 inhibition activity. This could be attributed to the change in the final conformation consequent to the fusion of Tubastatin A with Contilisant, which could have modified the ability of the new compounds to bind to the catalytic sites of different HDACs. Moreover, class I and class II HDACs share a region of around 390 amino acids that include the catalytic domain of the enzyme, and thus, it could be expected that such a compound could also affect other HDACs given the high similarity between them. In this regard, molecular docking considering the structure of the FRB compounds could help us better understand the reason behind the change in specificity and predict interactions between the compounds and different HDACs.

RNA-seq and proteomic analyses revealed that FRB44 or FRB56 treatment led to extensive transcriptional and proteomic remodelling, particularly affecting cell cycle- and synapse-related pathways. While genes/proteins within the cell cycle cluster are predominantly expressed by malignant cells (based on the GBMspace dataset), the expression of genes/proteins related to the synapse and vesicle clusters is broader, with neurons being the cell type exhibiting the highest expression levels. The integrated OMIC analyses demonstrate strong concordance between RNA and protein-level alterations, with significant correlations between cell cycle-related genes and FRB targets such as HDAC6 and BChE. These findings suggest that the antitumor effects of FRBs are mediated, at least in part, through modulation of epigenetic regulators controlling proliferation and cell cycle progression. In this regard, HDAC inhibitors are known to suppress tumor cell proliferation as well as to cause an arrest of the cell cycle by the regulation of the expression and activity of different cell cycle modulators and tumor suppressors [40]. Among them, p21^CIP^ and Ki67 are two of the most important targets [9,40], which we found significantly altered in both transcriptome and proteome approaches. Moreover, specific clusters of KI67 progenitor cells and neural progenitor or stem cells were downregulated in treated cells. Importantly, GNS models recapitulated the functional and molecular effects, reinforcing the relevance of FRB compounds in patient-derived stem cells and suggesting that these compounds are useful for tumor recurrence conditions. Moreover, OMIC studies also revealed upregulation of pathways more directly associated with neurological function, including regulation of neurotransmitters and synapses. These effects could be more directly linked to the inhibitory effect of MAOs and ChEs, as well as their potential modulation of H3R and S1R. These findings could be in line with the fact that contilisant was synthesized for neurodegenerative diseases, in order to improve neuronal activity.

Finally, our in vivo studies have shown potential antitumor effects of both compounds. However, it is important to acknowledge the inherent limitations of the subcutaneous model and U87 cells used in this study. Therefore, future studies must prioritize the use of orthotopic models and the incorporation of patient-derived cells in vivo to validate these findings. In this regard, it should be taken into account that we have validated the anti-proliferative effect of FRBs in different cell models and conditions in vitro through OMIC analyses and in vivo via immunohistochemistry. Moreover, we have demonstrated that both FRB44 and FRB56 substantially inhibited tumor growth without observable systemic toxicity. This is in agreement with the results obtained for the compound used as a starting point for the generation of the MSMs, Contilisant, as this compound presents no apparent adverse events and is able to cross the BBB [15]. Moreover, a recent study focused on testing two other MSMs generated by the juxtaposition of Contilisant and Tubastatin A showed them to be nontoxic in animal models [41].

Collectively, these results position FRB44 and FRB56 as promising candidates for glioblastoma treatment, combining favorable pharmacological properties with multimodal mechanisms of action. Their simultaneous targeting of HDACs, MAOs and ChE, along with their effects on cell cycle regulation, may offer advantages over single-target agents and address critical challenges in GBM therapy, including tumor heterogeneity and therapy resistance.

## 5. Patents

Some of the authors are part of the patent “Histone deacetylase derivatives for the treatment of cancer”, submission number: 300480865; application number: EP23382368.1. Authors: Marco Contelles, José Luis; Toledano Pinedo, Mireia; Porro Pérez, Alicia; Iturrioz Rodríguez, Nerea; Samprón Lebed, Nicolás; Artetxe Zurutuza, Aizpea; Matheu Fernández, Ander.

## Data Availability

The mass spectrometry proteomic data have been deposited to the Proteome Xchange Consortium via the PRIDE partner repository with the dataset identifier PXD069713. The RNA-Seq data have been deposited to the GEO repository with the dataset identifier GSE310919.

## Supplementary Materials

The following supporting information can be downloaded at: https://www.mdpi.com/article/10.3390/pharmaceutics17121594/s1. Figure S1: Characterization of the FRB compounds. (A,C,E,G) ^1^H NMR spectra of FRB21, FRB24, FRB44 and FRB56 respectively and (B,D,F,H) ^13^C NMR spectra of FRB21, FRB24, FRB44 and FRB56 respectively; Figure S2: Analysis of FRB compound targets’ expression on scRNA-seq data from GBMspace. (A) UMAP visualization detailing the more specific cellular subpopulations within the dataset. (B) Visualization of the expression levels of the FRB targets projected onto the UMAP space. (C) Dot plot illustrating the mean expression and the fraction of cells expressing each target gene in the broad Malignant and TME compartments. (D) Dot plot showing the mean expression and fraction of cells expressing the targets across the defined specific cellular states.; Figure S3: Inhibitory capacity of FRB44 and FRB56 of MAOs and BChE. (A) IC50 values of FRBs and reference compounds Clorgiline Selegiline and Rasalgiline as MAO-A and MAO-B inhibitors. (B) IC50 values of FRBs and reference compounds Donepezil and Rivastigmine as BChE inhibitors. (C) Lineweaver-Burk plot representing reciprocal of velocity versus reciprocal of substrate concentrations at different concentrations of FBR44 and (D) Secondary plot of slopes obtained in Lineweaver-Burk plot versus different inhibitor concentrations for the estimation of Ki for FRB44; Figure S4: FRB44 and FRB56 are cytotoxic for glioblastoma, glioma and patient-derived GSCs. (A,B) Quantification and representative pictures of p-H3 positive, (C,D) Ki67 positive and (E,F) Cleaved caspase-3 cells after 1, 3 and 5 µM of FRB44, FRB56 and Tubastatin A treatments in U87 cells. Scale bar 200 μM, immunofluorescence images are included after 5 μM of treatment. (G) Quantification of pHH3 positive and (H) cleaved caspase-3 positive cells after FRB44 and FRB56 treatment in the GNS179 and I-J) U251 cell lines; Figure S5: PCA plots of (A) RNA-seq and (B) proteomics of FRB44 and FRB56; Figure S6: (A,B) Gene ontology study of RNA-seq representing the 20 most correlated pathways with upregulated and downregulated DEGs after FRB44 and FRB56 treatments; Figure S7: (A,B) Gene ontology study of proteomics representing the 20 most correlated pathways with upregulated and downregulated DEGs after FRB44 and FRB56 treatments; Figure S8: (A) GSEA of senescence signatures and associated pathways derived from the Molecular Signatures Database (MSigDB). (B) ORA of DEPs identified in the FRB44 proteomics dataset, annotated with brain-specific cell types sourced from the CellMarker database; Figure S9: (A,B) Gene ontology terms and corresponding numbers of the clusters showed in Figure 5A and 5B after FRB44 and FRB56 treatments, respectively; Figure S10: (A,B) Spearman correlation analysis of common DEGs and DEPs cell cycle clusters obtained from FRB44 and FRB56 omics with target genes in TCGA cohort; Figure S11: Analysis of FRB Compound Target Expression Signatures in GBM Space scRNA-seq Data. (A) Uniform Manifold Approximation and Projection (UMAP) visualization and associated heatmap depicting the mean module scores derived from the genes commonly differentially expressed (DEGs) within each cluster defined by the FRB44 multi-omic analysis. (B) UMAP visualization and heatmap of the mean module scores based on the common DEGs identified within each cluster from the FRB56 multi-omic analysis.
