# Supplementary material for: Generation and Characterization of Novel Contilisant+Tubastatin a Multitarget Small Molecules Against Glioblastoma
Source: Pharmaceutics. 2025 Dec 10;17(12):1594. doi: 10.3390/pharmaceutics17121594 (PMC12736535; doi:10.3390/pharmaceutics17121594)
Supplement: Supplementary file 1 [file pharmaceutics-17-01594-s001.zip › pharmaceutics-3981336-supplementary.pdf]

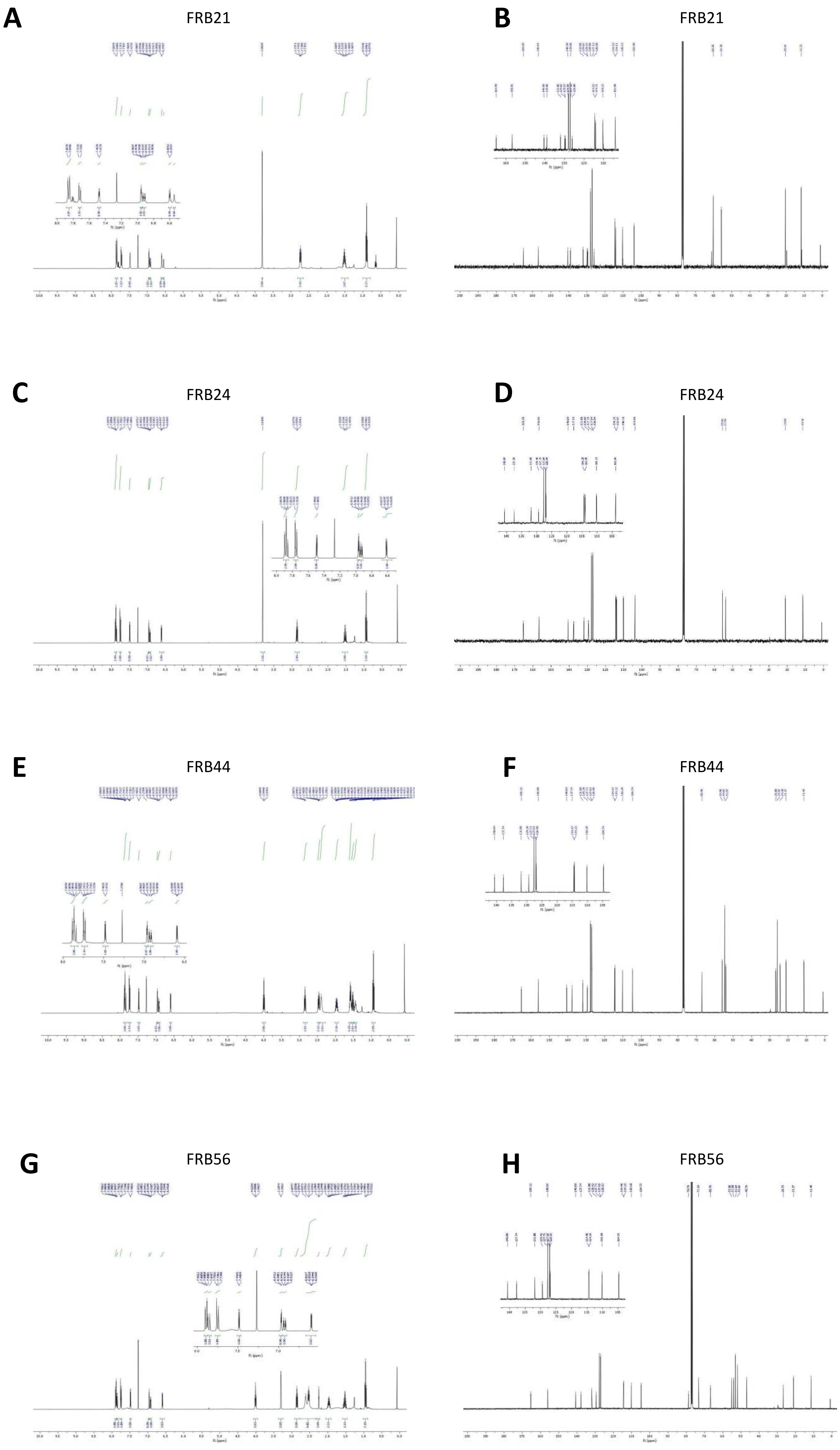

Figure S1: Characterization of the FRB compounds. (A,C,E,G) ^{1}H NMR spectra of FRB21, FRB24, FRB44 and FRB56 respectively and (**B,D,F,H)** ^{13}C NMR spectra of FRB21, FRB24, FRB44 and FRB56 respectively.

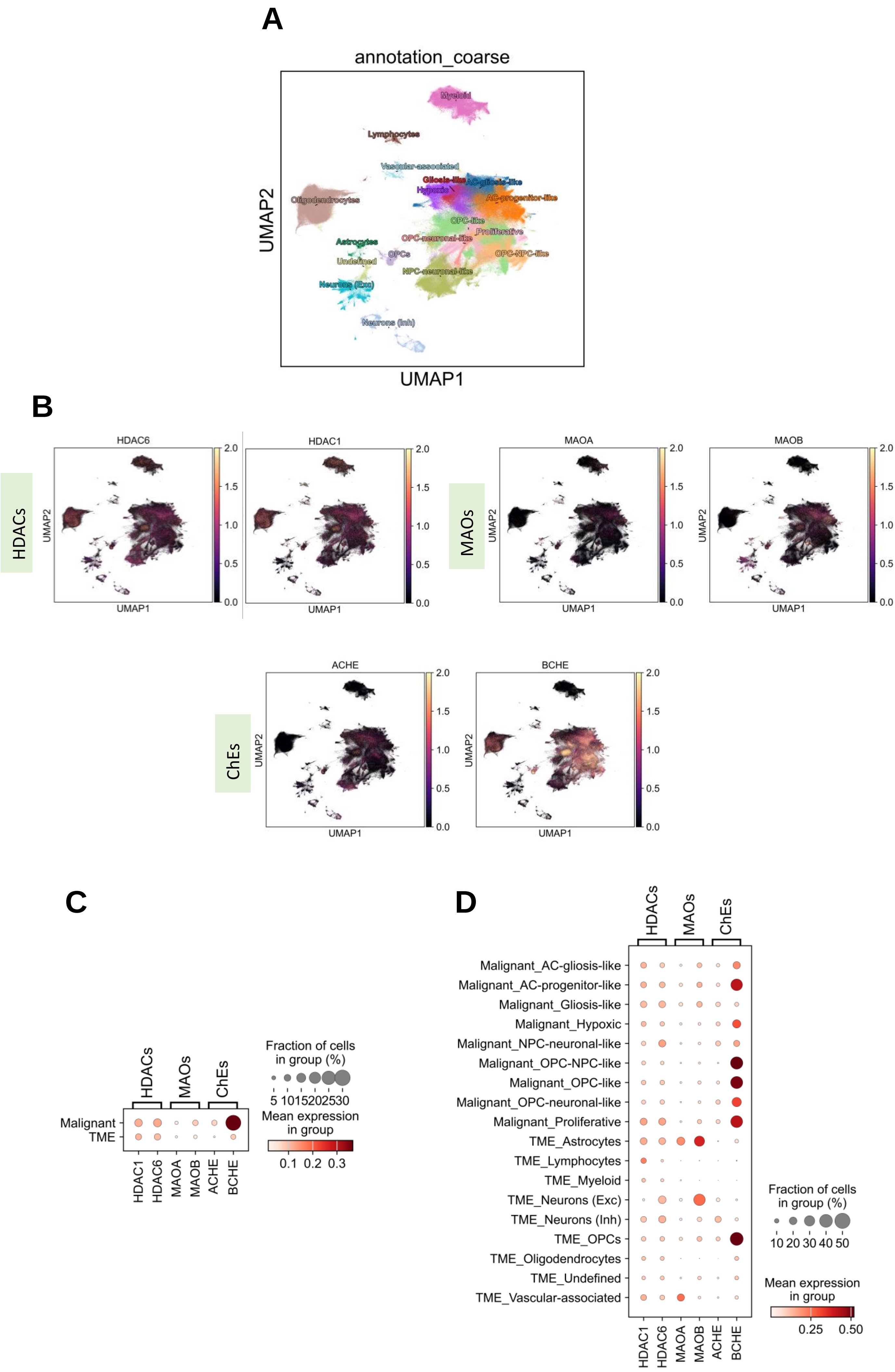

Suppl. Fig.2: Analysis of FRB compound targets' expression on scRNA-seq data from GBMspace. (A) UMAP visualization detailing the more specific cellular subpopulations within the dataset. **(B)** Visualization of the expression levels of the FRB targets projected onto the UMAP space. **(C)** Dot plot illustrating the mean expression and the fraction of cells expressing each target gene in the broad Malignant and TME compartments. **(D)** Dot plot showing the mean expression and fraction of cells expressing the targets across the defined specific cellular states.

A

MAO inhibition

Compound	**MAO-A %** IC50 μM	**MAO-B %** IC50 μM
FRB44	-	3.16
FRB56	1.68	0.15
Clorgiline	$9.66x10^{-4}$	-
Selegiline	-	$2.86x10^{-3}$
Rasalgiline	-	$4.93x10^{-3}$

B

ChE inhibition

Compound	**BChE %** IC50 μM
FRB44	8.24
FRB56	-
Rovastigmine	0.012

C

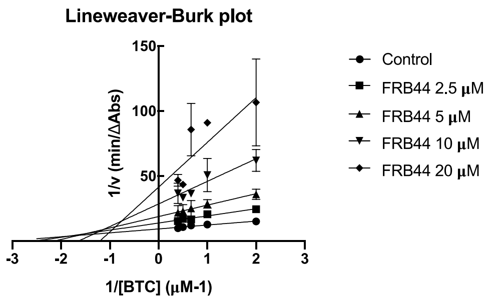

	Type of inhibition
FRB44	Mixed

D

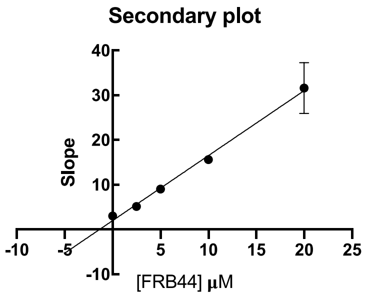

	Ki (μM)
FRB44	1.36

Figure S3: Inhibitory capacity of FRB44 and FRB56 of MAOs and BChE. (**A)** IC50 values of FRBs and reference compounds Clorgiline Selegiline and Rasalgiline as MAO-A and MAO-B inhibitors. (**B)** IC50 values of FRBs and reference compounds Donepezil and Rivastigmine as BChE inhibitors. (**C)** Lineweaver-Burk plot representing reciprocal of velocity versus reciprocal of substrate concentrations at different concentrations of FBR44 and (**D)** Secondary plot of slopes obtained in Lineweaver-Burk plot versus different inhibitor concentrations for the estimation of Ki for FRB44.

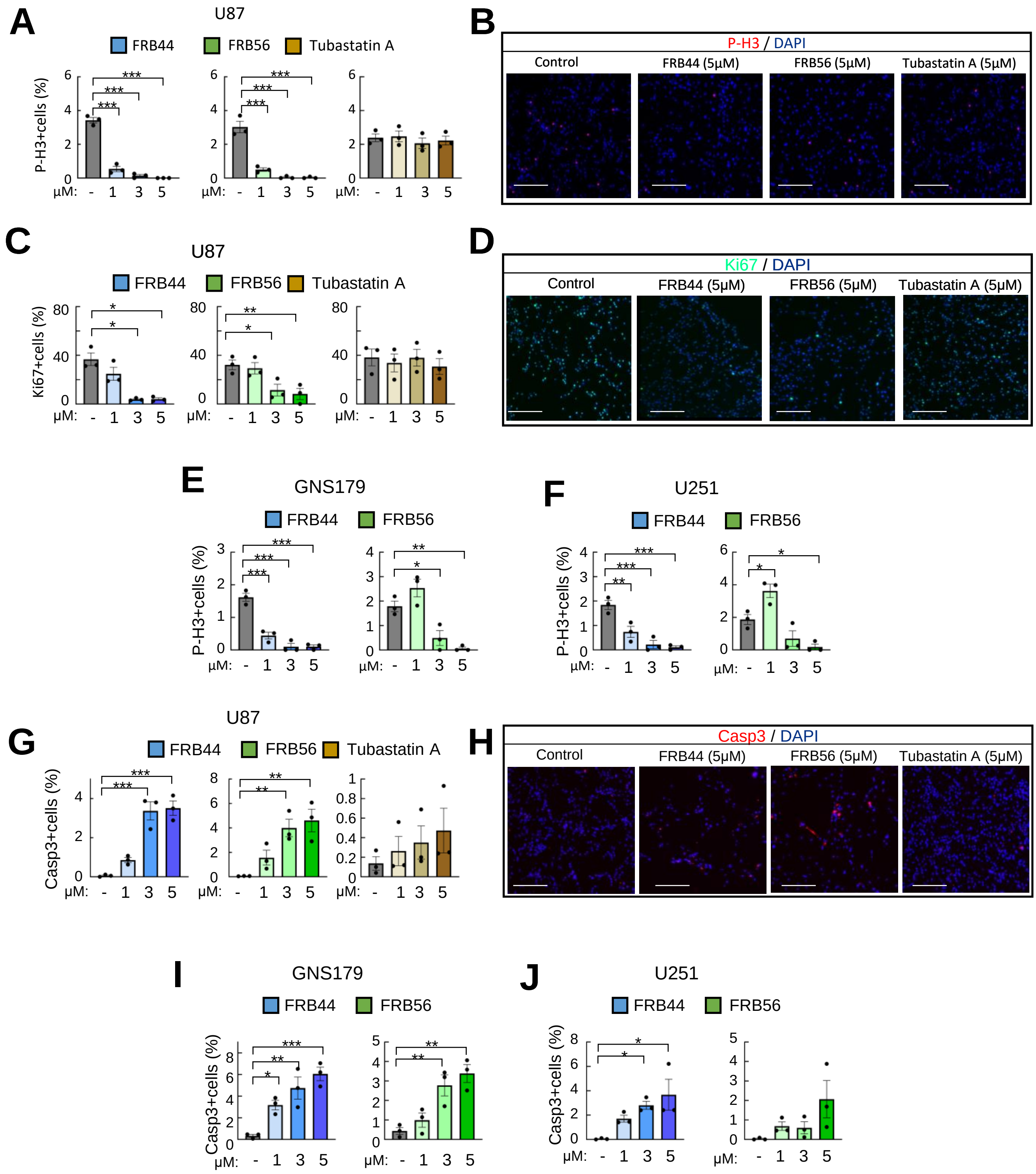

Figure S4: FRB44 and FRB56 are cytotoxic for glioblastoma, glioma and patient-derived GSCs. (A-B) Quantification and representative pictures of p-H3 positive, (**C-D)** Ki67 positive and (**E-F)** Cleaved caspase-3 cells after 1, 3 and 5 µM of FRB44, FRB56 and Tubastatin A treatments in U87 cells. Scale bar 200µM, immunofluorescence images are included after 5µM of treatment. (**G)** Quantification of pHH3 positive and (**H)** cleaved caspase-3 positive cells after FRB44 and FRB56 treatment in the GNS179 and (**I-J)** U251 cell lines.

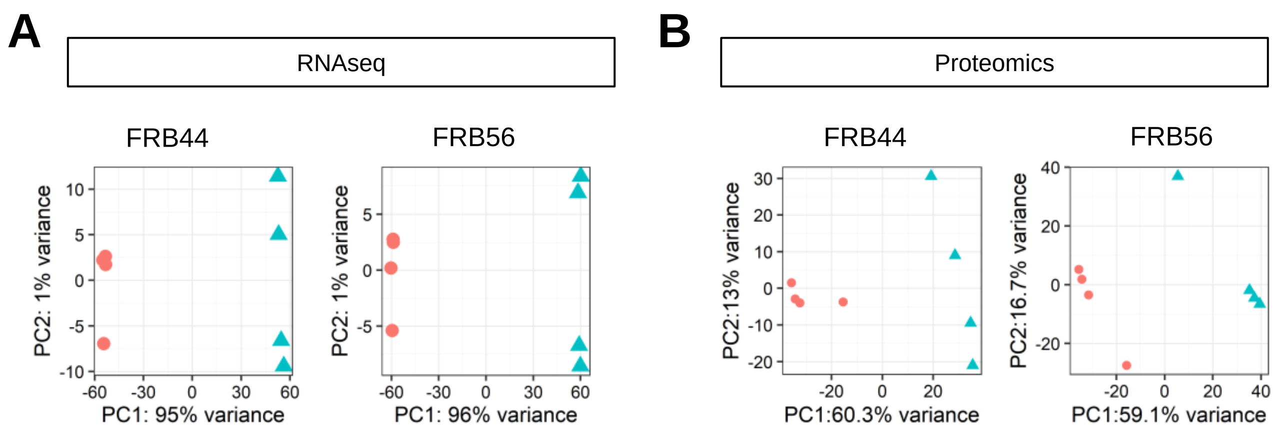

Figure S5: PCA plots of RNA-seq (A) and proteomics (B) of FRB44 and FRB56

A

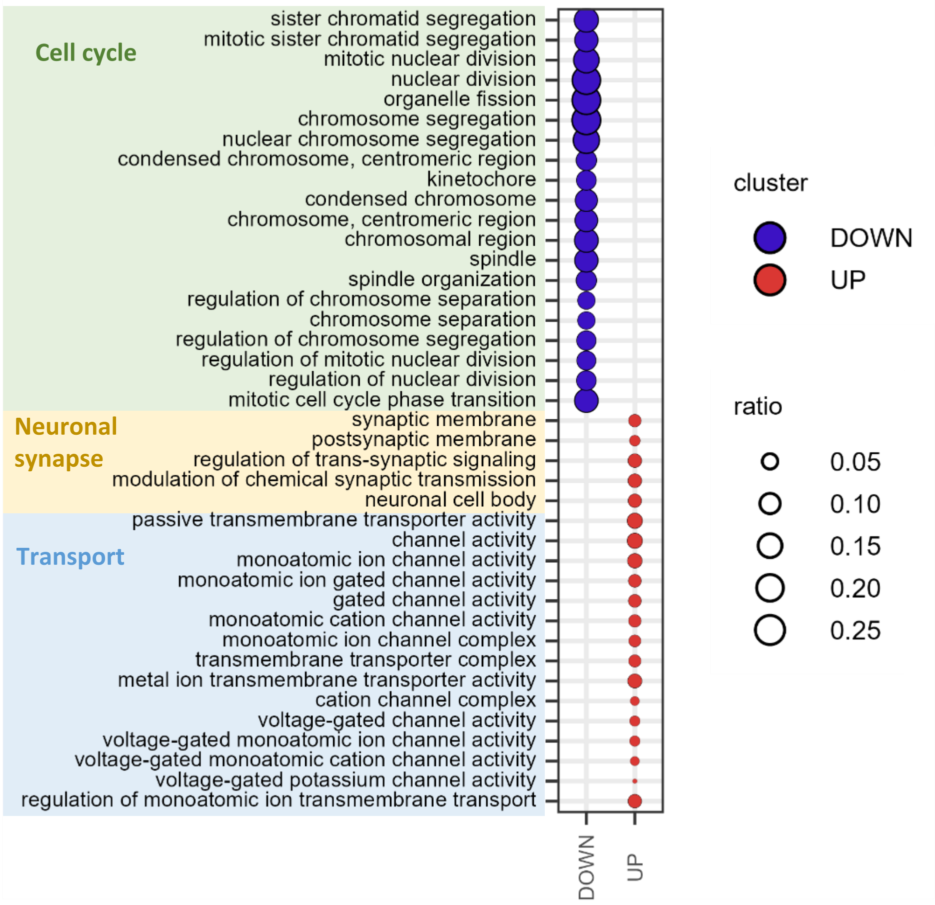

B

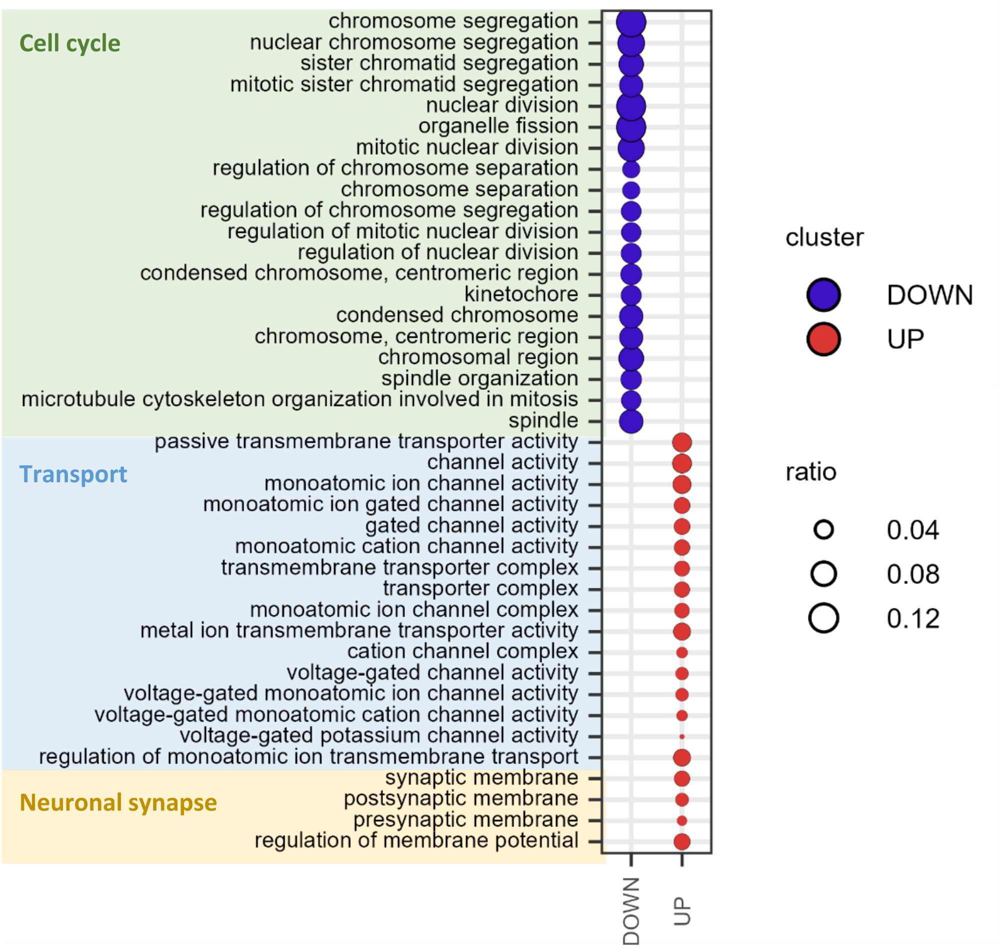

Figure S6: **(A-B)** Gene ontology study of RNA-seq representing the 20 most correlated pathways with upregulated and downregulated DEGs after FRB44 and FRB56 treatments.

A

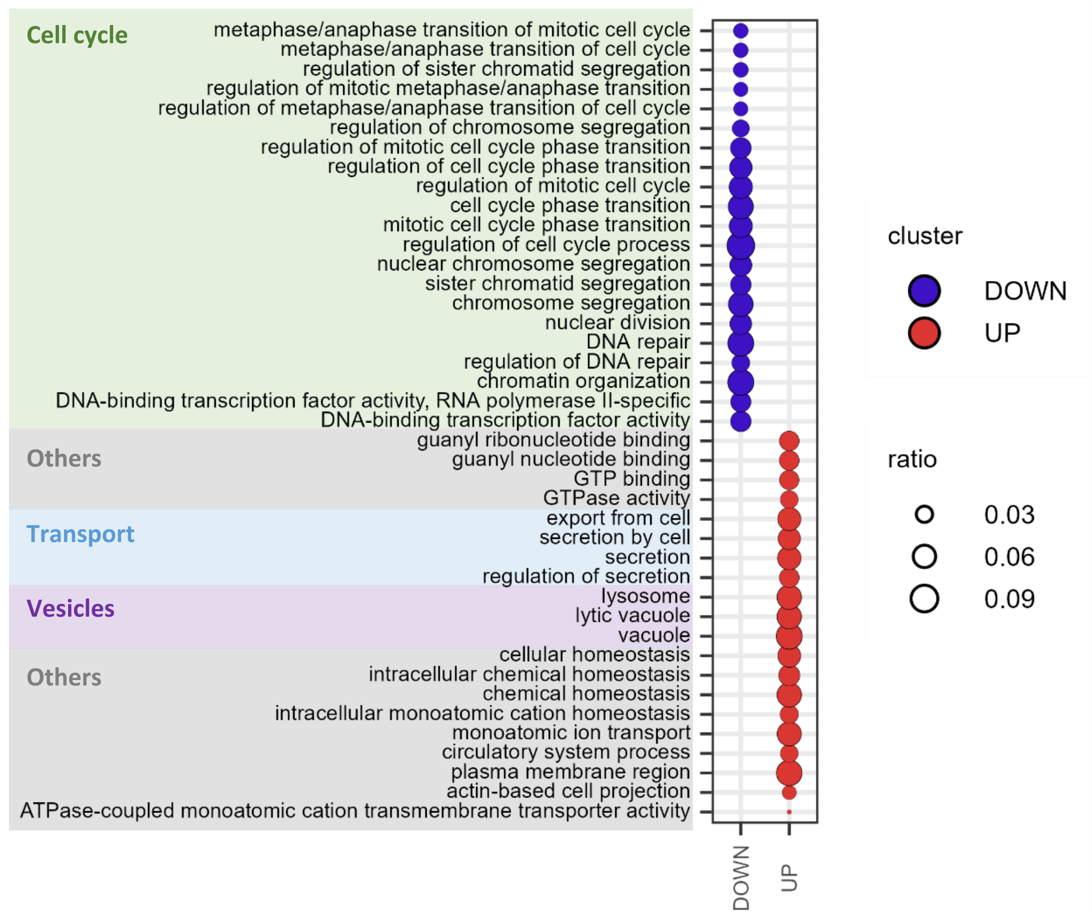

B

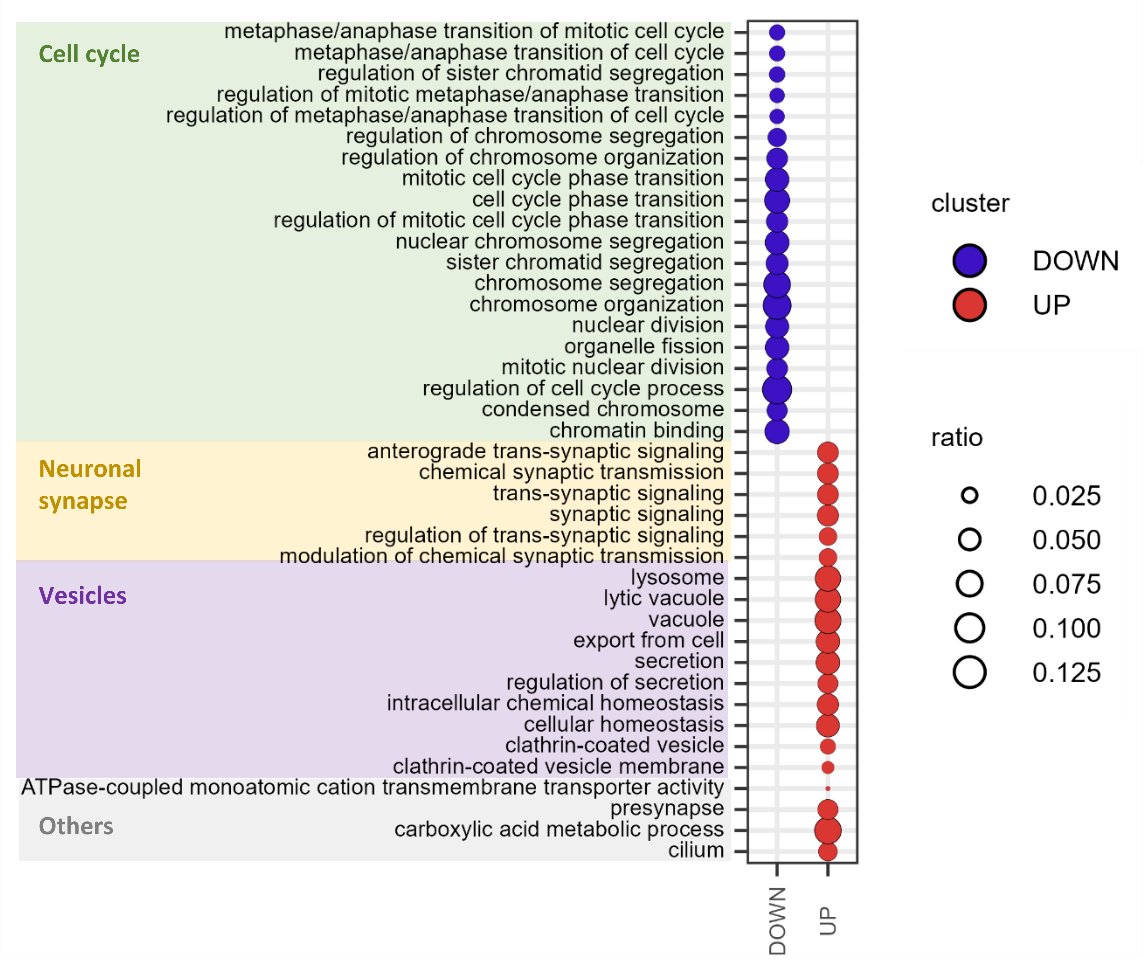

Figure S7: **(A-B)** Gene ontology study of proteomics representing the 20 most correlated pathways with upregulated and downregulated DEGs after FRB44 and FRB56 treatments.

GSEA of senescence-related processes

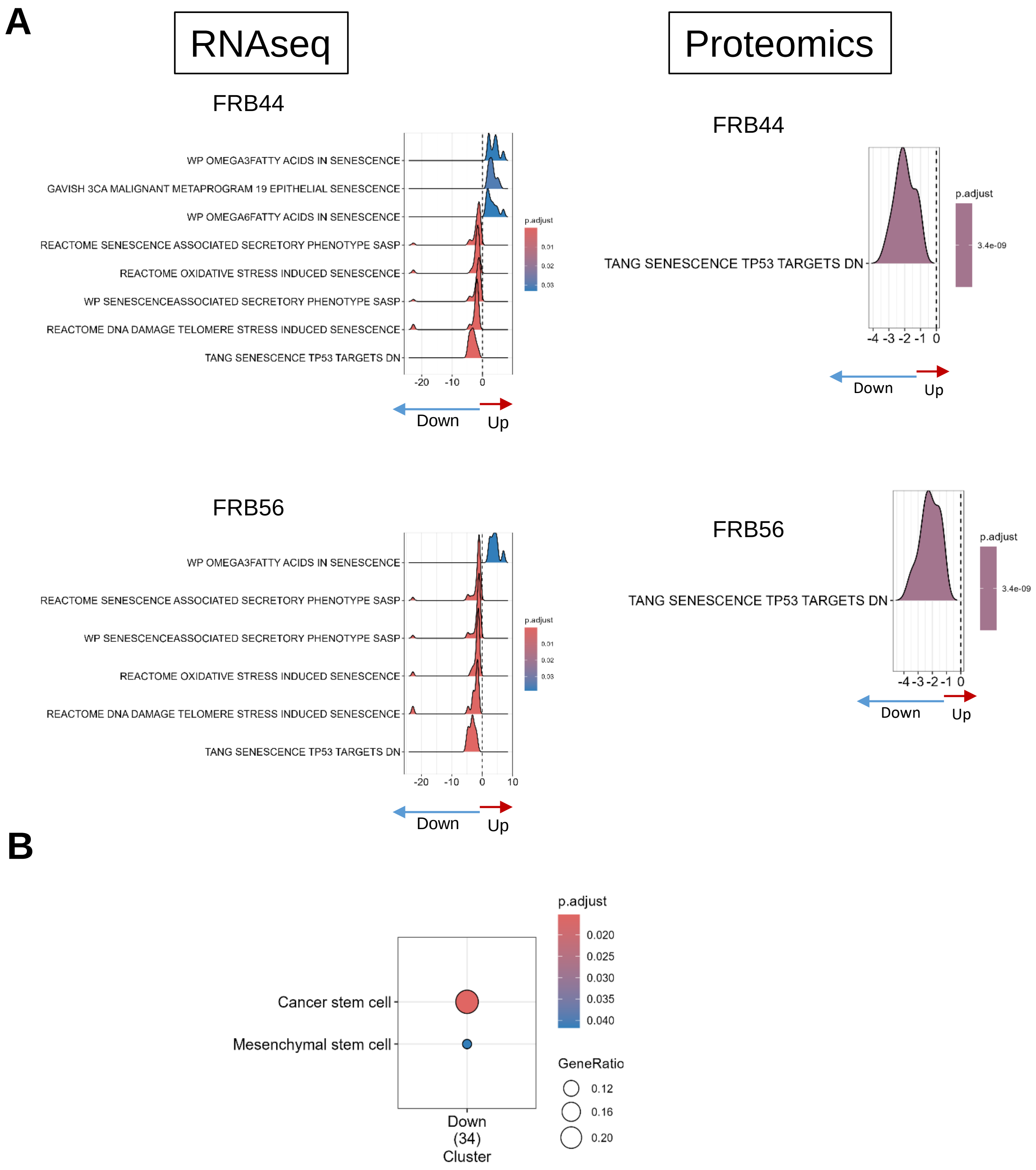

Figure S8: (A) GSEA of senescence signatures and associated pathways derived from the Molecular Signatures Database (MSigDB). (**B)** ORA of DEPs identified in the FRB44 proteomics dataset, annotated with brain-specific cell types sourced from the CellMarker database.

A

Cell cycle

1 DNA replication
2 DNA-binding transcription factor binding
3 nuclear chromosome
4 transcription coactivator activity
5 histone modification
6 chromatin remodeling
7 peptidyl-lysine modification
8 SWI/SNF superfamily-type complex
9 centromere complex assembly
10 regulation of DNA repair
11 histone acetyltransferase complex
12 double-strand break repair
13 condensed chromosome
14 kinetochore organization
15 spindle assembly checkpoint signaling
16 metaphase/anaphase transition of mitotic cell cycle
17 chromosome, centromeric region
18 regulation of chromosome organization
19 regulation of chromosome segregation
20 sister chromatid segregation
21 chromosome segregation
22 regulation of cell cycle phase transition
23 chromosome localization
24 nuclear chromosome segregation
25 regulation of chromosome separation
26 mitotic cell cycle phase transition
27 nuclear division
28 regulation of mitotic nuclear division
29 mitotic nuclear division
30 cell cycle checkpoint signaling
31 spindle organization
32 regulation of nuclear division
33 microtubule binding
34 mitotic spindle checkpoint signaling
35 cell cycle G2/M phase transition
36 non-membrane-bounded organelle assembly
37 spindle
38 microtubule motor activity
39 mitotic cytokinesis
40 regulation of cell division
41 midbody

Transport

1 potassium ion transport
2 import into cell
3 inorganic cation import across plasma membrane
4 import across plasma membrane
5 potassium ion transmembrane transport
6 inorganic ion import across plasma membrane
7 metal ion transmembrane transporter activity
8 passive transmembrane transporter activity
9 calcium ion transport
10 transporter complex
11 regulation of monoatomic ion transmembrane transport
12 monoatomic ion channel activity
13 regulation of membrane potential
14 monoatomic ion channel complex

Vesicle

1 pigment granule
2 establishment of organelle localization
3 cellular component disassembly
4 melanosome
5 vesicle organization
6 vacuolar membrane
7 macroautophagy
8 ATPase-coupled monoatomic cation transmembrane transporter activity
9 regulation of macroautophagy
10 vacuole organization
11 vacuolar transport
12 cytosolic transport

Synapse

1 synaptic membrane
2 signal release
3 regulation of trans-synaptic signaling
4 regulation of neurotransmitter levels
5 modulation of chemical synaptic transmission
6 neuron to neuron synapse
7 neurotransmitter transport
8 vesicle-mediated transport in synapse
9 neuronal cell body

Cell adhesion

1 actin-based cell projection
2 cell leading edge
3 actin binding
4 cell-substrate junction

GTPase activity

1 GTP binding
2 guanyl nucleotide binding
3 guanyl ribonucleotide binding
4 GTPase activity

B

Cell cycle

1 microtubule motor activity
2 non-membrane-bounded organelle assembly
3 microtubule binding
4 midbody
5 chromosome localization
6 chromosome, centromeric region
7 spindle
8 chromosome segregation
9 chromosomal region
10 nuclear division
11 nuclear chromosome segregation
12 sister chromatid segregation
13 regulation of cell division
14 spindle organization
15 mitotic nuclear division
16 regulation of chromosome organization
17 regulation of chromosome segregation
18 mitotic cytokinesis
19 regulation of chromosome separation
20 metaphase/anaphase transition of mitotic cell cycle
21 regulation of nuclear division
22 regulation of mitotic nuclear division
23 mitotic cell cycle phase transition
24 regulation of mitotic cell cycle phase transition
25 spindle assembly checkpoint signaling
26 mitotic spindle checkpoint signaling
27 cell cycle G2/M phase transition
28 DNA replication

Organic acid catabolism

1 organic acid catabolic process
2 mitochondrial matrix
3 small molecule catabolic process
4 carboxylic acid catabolic process

DNA remodelling

1 nuclear matrix
2 chromatin remodeling
3 double-strand break repair
4 peptidyl-lysine modification
5 regulation of DNA repair
6 histone modification
7 SWI/SNF superfamily-type complex
8 transcription coactivator activity

Vesicle

1 primary lysosome
2 azurophil granule
3 cytosolic transport
4 lysosomal transport
5 lytic vacuole membrane
6 lysosomal membrane
7 vacuole organization
8 vacuolar membrane
9 clathrin-coated vesicle
10 macroautophagy
11 clathrin-coated vesicle membrane
12 cellular component disassembly
13 regulation of macroautophagy
14 synaptic vesicle cycle
15 ATPase-coupled monoatomic cation transmembrane transporter activity
16 synaptic vesicle maturation

Synapse

1 synaptic membrane
2 vesicle-mediated transport in synapse
3 neurotransmitter transport
4 regulation of trans-synaptic signaling
5 glutamatergic synapse
6 regulation of neurotransmitter levels
7 modulation of chemical synaptic transmission
8 signal release
9 neuronal cell body0

Transport

1 vascular process in circulatory system
2 calcium ion transport
3 import into cell
4 inorganic cation import across plasma membrane
5 inorganic ion import across plasma membrane
6 muscle contraction
7 import across plasma membrane
8 metal ion transmembrane transporter activity
9 regulation of monoatomic ion transmembrane transport
10 transporter complex
11 monoatomic ion channel activity
12 potassium ion transport
13 monoatomic ion channel complex
14 passive transmembrane transporter activity
15 regulation of membrane potential

Figure S9: (A-B) Gene ontology terms and corresponding numbers of the clusters showed in **Figure 5A** and **5B** after FRB44 and FRB56 treatments, respectively.

A

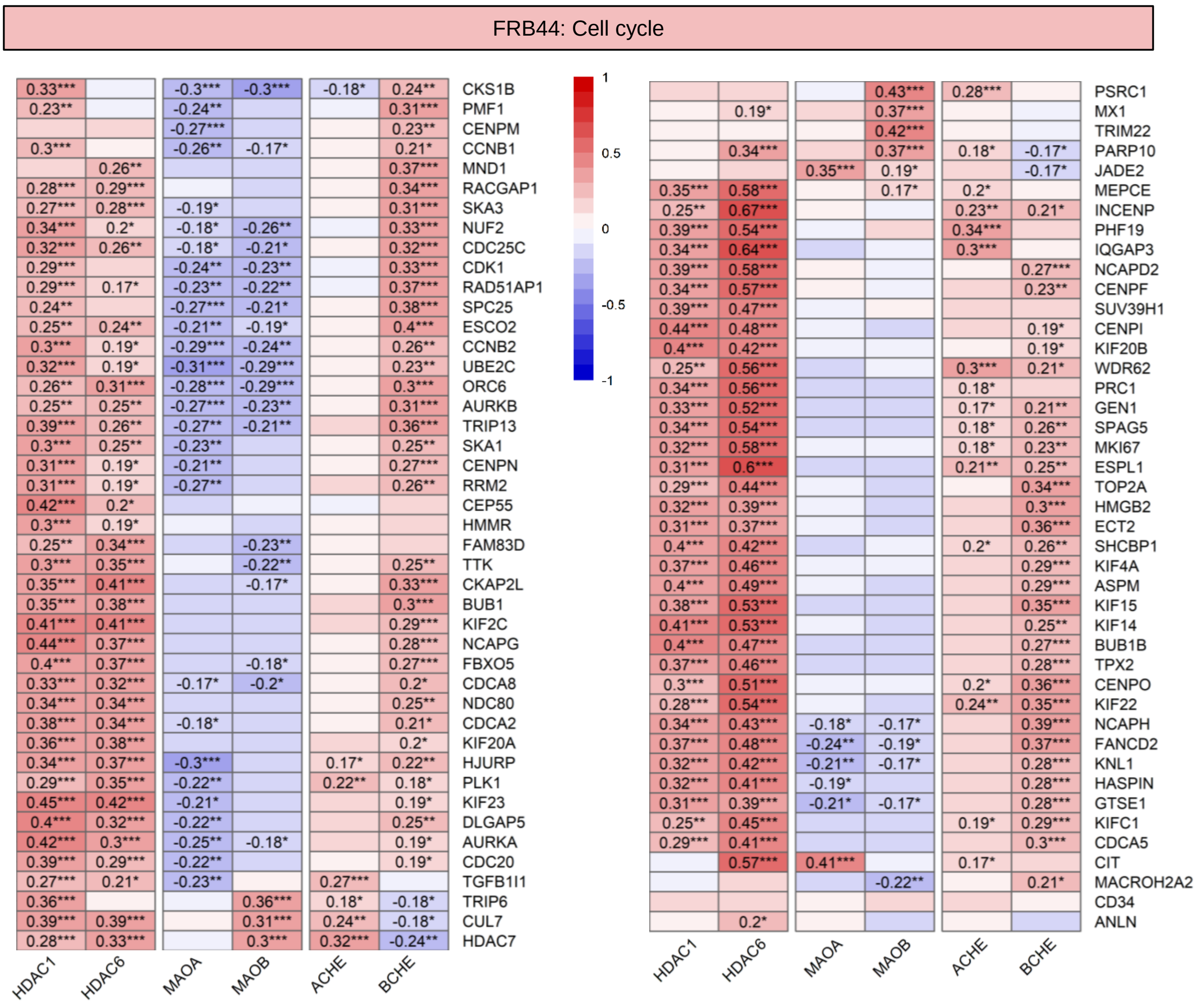

B

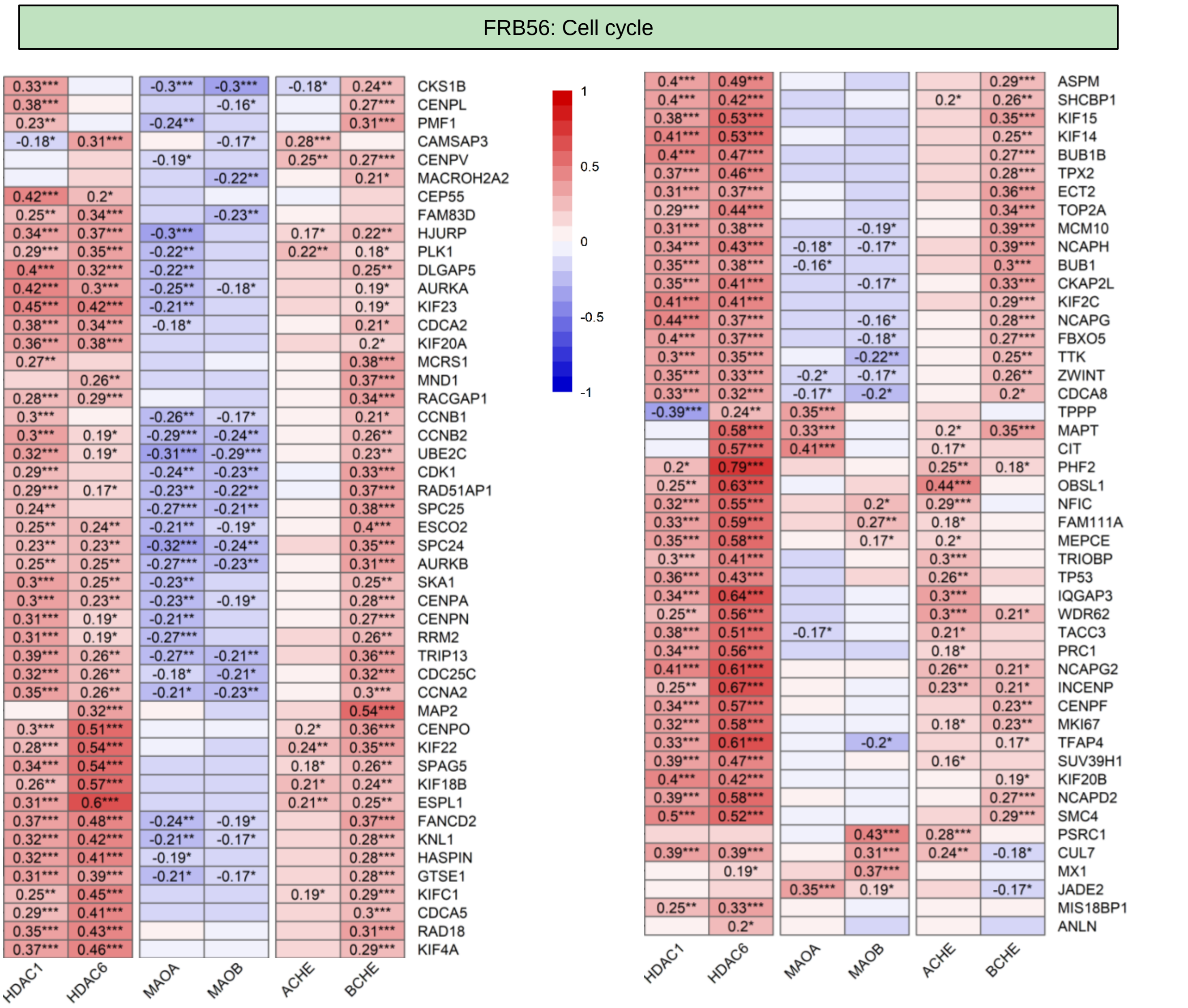

Figure S10: (A-B) Spearman correlation analysis of common DEGs and DEPs cell cycle clusters obtained from FRB44 and FRB56 omics with target genes in TCGA cohort.

A

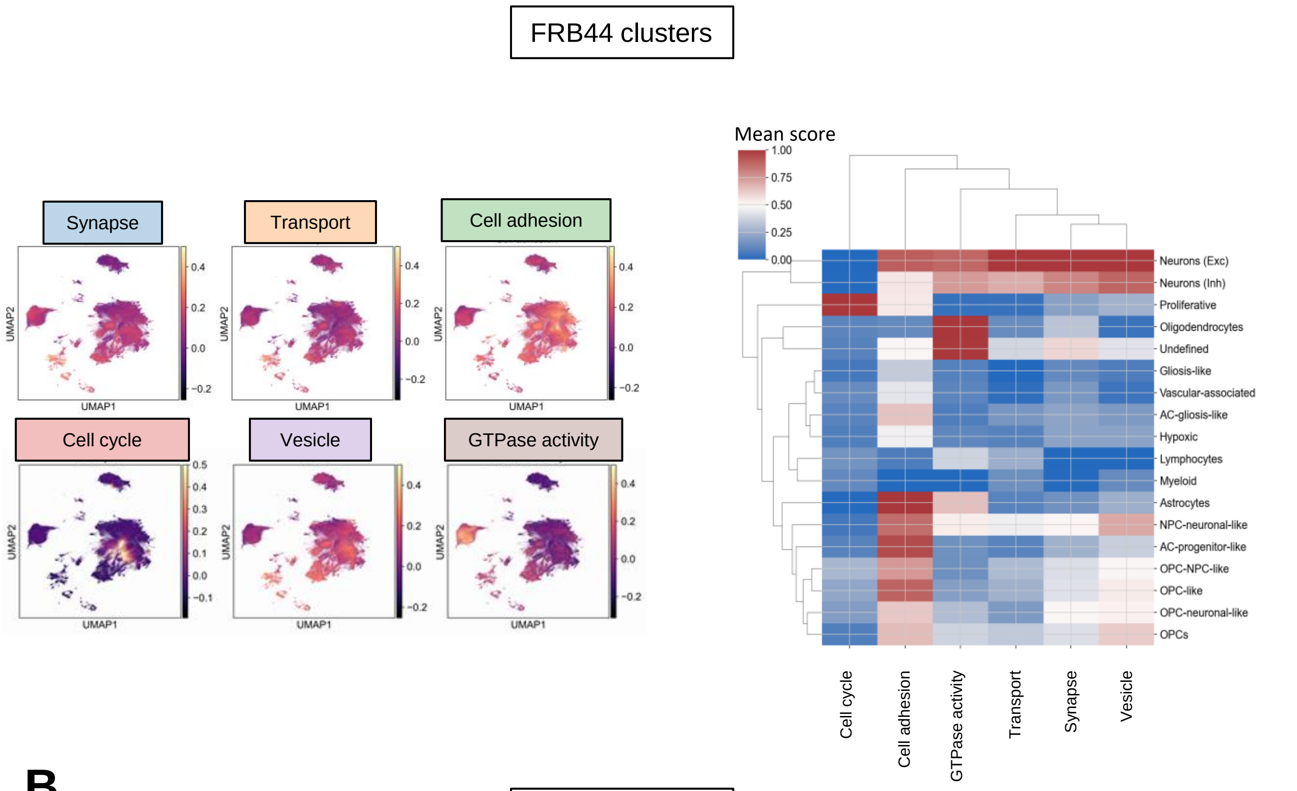

B

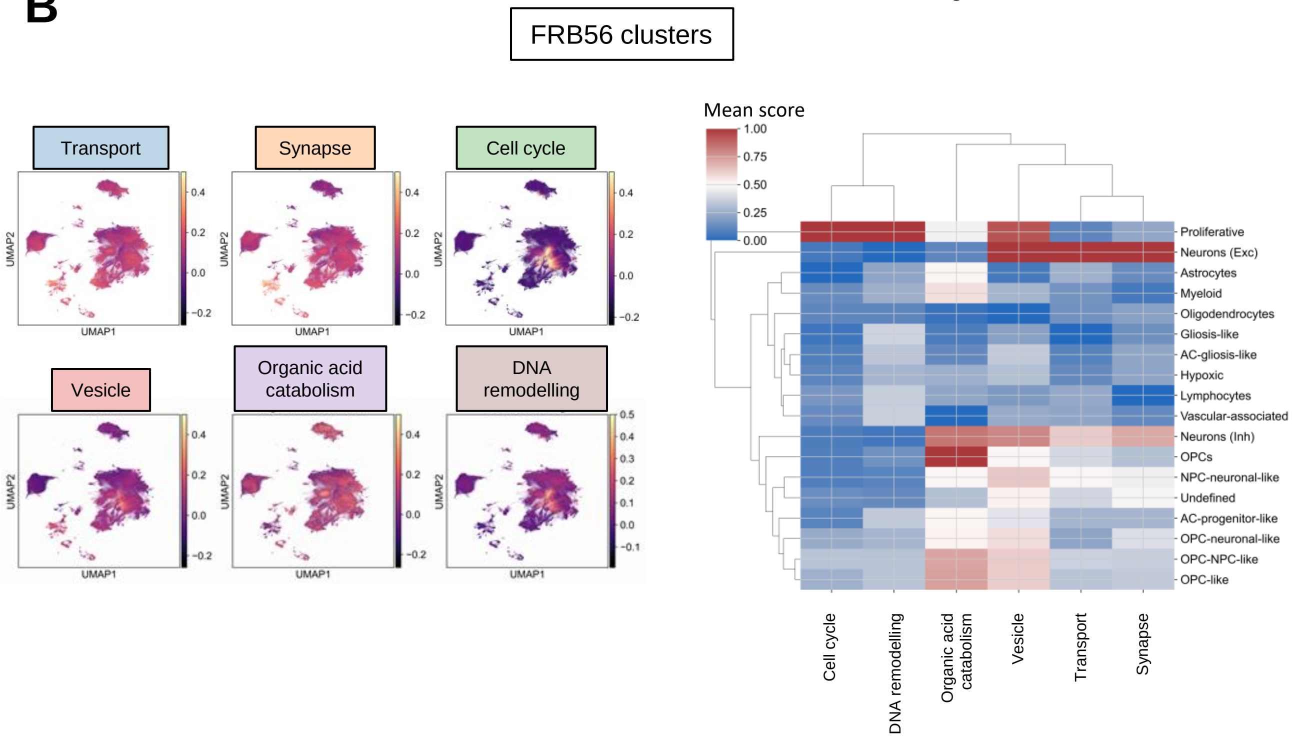

Figure S11: Analysis of FRB Compound Target Expression Signatures in GBM Space scRNA-seq Data. (A) Uniform Manifold Approximation and Projection (UMAP) visualization and associated heatmap depicting the mean module scores derived from the genes commonly differentially expressed (DEGs) within each cluster defined by the FRB44 multi-omic analysis. **(B)** UMAP visualization and heatmap of the mean module scores based on the common DEGs identified within each cluster from the FRB56 multi-omic analysis.
